# Positive feedback loop of c-myc/XTP6/NDH2/NF-κB to promote malignant progression in glioblastoma

**DOI:** 10.1186/s13046-024-03109-5

**Published:** 2024-07-05

**Authors:** Feng Xiao, Hong Zhu, Yaping Xiong, Yun Guo, Zhe Zhang, Jie Zeng, Yao Xiao, Bin Liao, Xuesong Shang, Siyi Zhao, Guowen Hu, Kai Huang, Hua Guo

**Affiliations:** 1 Department of Neurosurgery, The Second Affiliated Hospital, Jiangxi Medical College, Nanchang University, Nanchang, Jiangxi 330006 China; 2 Jiangxi Key Laboratory of Neurological Tumors and Cerebrovascular Diseases, Nanchang University, Nanchang, Jiangxi 330006 China; 3 JXHC key Laboratory of Neurological medicine, Nanchang University, Nanchang, Jiangxi 330006 China; 4 Institute of Neuroscience, Nanchang University, Nanchang, Jiangxi 330006 China; 5 Jiangxi Province Key Laboratory of Neurological Diseases, Nanchang University, Nanchang, Jiangxi 330006 China; 6 Departments of Anesthesiology, The Second Affiliated Hospital, Jiangxi Medical College, Nanchang University, Nanchang, Jiangxi 330006 China

**Keywords:** Positive feedback loop, Glioblastoma, LncRNA, XTP6, NF-κB signaling pathway

## Abstract

**Background:**

Recent studies have highlighted the significant role of the NF-κB signaling pathway in the initiation and progression of cancer. Furthermore, long noncoding RNAs (lncRNAs) have been identified as pivotal regulators in sustaining the NF-κB signaling pathway’s functionality. Despite these findings, the underlying molecular mechanisms through which lncRNAs influence the NF-κB pathway remain largely unexplored.

**Methods:**

Bioinformatic analyses were utilized to investigate the differential expression and prognostic significance of XTP6. The functional roles of XTP6 were further elucidated through both in vitro and *in vivo* experimental approaches. To estimate the interaction between XTP6 and NDH2, RNA pulldown and RNA Immunoprecipitation (RIP) assays were conducted. The connection between XTP6 and the IκBα promoter was examined using Chromatin Isolation by RNA Purification (ChIRP) assays. Additionally, Chromatin Immunoprecipitation (ChIP) assays were implemented to analyze the binding affinity of c-myc to the XTP6 promoter, providing insights into the regulatory mechanisms at play.

**Results:**

XTP6 was remarkedly upregulated in glioblastoma multiforme (GBM) tissues and was connected with adverse prognosis in GBM patients. Our investigations revealed that XTP6 can facilitate the malignant progression of GBM both in vitro and in vivo. Additionally, XTP6 downregulated IκBα expression by recruiting NDH2 to the IκBα promoter, which resulted in elevated levels of H3K27me3, thereby reducing the transcriptional activity of IκBα. Moreover, the progression of GBM was further driven by the c-myc-mediated upregulation of XTP6, establishing a positive feedback loop with IκBα that perpetuated the activation of the NF-κB signaling pathway. Notably, the application of an inhibitor targeting the NF-κB signaling pathway effectively inhibited the continuous activation induced by XTP6, leading to a significant reduction in tumor formation in vivo.

**Conclusion:**

The results reveal that XTP6 unveils an innovative epigenetic mechanism instrumental in the sustained activation of the NF-κB signaling pathway, suggesting a promising therapeutic target for the treatment of GBM.

**Supplementary Information:**

The online version contains supplementary material available at 10.1186/s13046-024-03109-5.

## Introduction

Glioblastoma (GBM) is recognized as the most aggressive and common primary brain tumor among adults [1, 2]. Despite the implementation of intensive treatment protocols, such as comprehensive surgical resection complemented by chemotherapy and radiotherapy, the outlook for individuals diagnosed with GBM continues to be dire. This grim prognosis is primarily attributed to the emergence of therapeutic resistance and the recurrence of the tumor following surgery, with the median survival duration being less than 15 months [3, 4]. Despite the emergence of novel therapeutic approaches in recent years, such as immunotherapy, electric field therapy, and targeted therapy, the recurrence of GBM in patients remains common due to the blood-brain barrier, the infiltrative nature of the tumor, and its unique immune microenvironment [5–7]. Consequently, it is imperative to delve into the distinct molecular underpinnings of GBM. Understanding these mechanisms will facilitate the creation of innovative therapeutic agents for GBM management, ultimately enhancing the survival prospects of patients with this condition.

LncRNAs are a category of RNA molecules, extending beyond 200 nucleotides in length, which do not possess the ability to translate into proteins [8, 9]. Extensive research has elucidated that lncRNAs serve as crucial regulators of gene expression, engage in intricate regulatory interactions with tumor-associated gene expression across epigenetic, transcriptional, and post-transcriptional stages, and are intimately linked to the initiation of tumorigenesis and its malignant progression [10–12]. LncRNAs, through their specific cellular localization and unique interactions with proteins, DNA, and RNA, can modulate chromatin function, regulate the stability and translation of mRNA in the cytoplasm, and intervene in various signaling pathways, thereby promoting the malignant progression of cancer [13–16].

Multiple investigations have revealed a significant correlation between the activation of the NF-κB signaling pathway and the malignant progression of cancer [17–19]. Upon cytokine stimulation, the inhibitory κB (IκB) is phosphorylated by the activated IκB kinase (IKK) complex, leading to the ubiquitination and subsequent degradation of inhibitory κBα (IκBα). Consequently, NF-κB, which is sequestered by IκB in the cytoplasm, is liberated and translocates to the nucleus, thus initiating the transcriptional activation of diverse genes [20]. Numerous studies have demonstrated that lncRNAs promote malignant progression of cancer by activating NF-κB signaling pathway [21–24]. For instance, PTRF, identified as a unique RNA-interacting protein, accelerated the NF-κB/PD-L1 pathway by stabilizing lncRNA NEAT1, facilitating tumor development and immune escape in GBM [25]. LncRNA SChLAP1 engaged in a complex formation with HNRNPL, effectively ensuring the stability of ACTN4 expression and the activation of the NF-κB signaling pathway, thus accelerating the malignant progression of GBM [26]. Despite numerous lncRNAs have been identified in GBM, the mechanisms underlying their regulation of the NF-κB signaling pathway remain incompletely understood.

LncRNA XTP6, alternatively termed deleted in lymphocytic leukemia 1, is situated on chromosome 13q14.3. In this research, we revealed that XTP6 expression was elevated in GBM tissues and correlated positively with an unfavorable prognosis in GBM patients. The data showed that silencing XTP6 led to a reduction in the malignant progression of GBM, both in vitro and in vivo. Additionally, our findings verified that XTP6 facilitated the activation of the NF-κB signaling pathway through the downregulation of IκBα expression. Moreover, XTP6 was instrumental in the continuous activation of the NF-κB pathway through creating a positive feedback loop with the transcription factor c-myc.

## Materials and methods

### GBM data acquisition

Two independent GBM datasets—CGGA (CGGA_325) and GSE16011—were utilized in the study. The gene expression profiles, and survival statistics were collected from the Chinese Glioma Genomic Atlas (CGGA, http://www.cgga.org.cn/) and the Gene Expression Omnibus (GEO, https://www.ncbi.nlm.nih.gov/gds) databases. Additionally, the pan-cancer analysis of XTP6 was conducted on the SangerBox website (https://www.sangerbox.com/).

### Clinical samples collection

Between 2018 and 2023, samples from twelve GBM patients, along with para-cancerous tissues (PCTs), were surgically excised under the auspices of the Department of Neurosurgery at the Second Affiliated Hospital, Nanchang University. To ensure the integrity of RNA and protein analyses, tissue specimens were immediately immersed in liquid nitrogen post-excision. For immunohistochemistry (IHC) analysis, these specimens underwent a preservation process, initially being fixed in 10% neutral-buffered formalin, followed by dehydration with 70% ethanol, and ultimately embedded in paraffin. Informed consents for participation in this study were obtained from the patients with GBM. The research protocol was granted ethical clearance by the Medical Ethics Committee at the Second Affiliated Hospital, Nanchang University (NO. Review [2021] NO. (033)).

### RNA in situ hybridization (RNA-ISH) and IHC assays

*RNA-ISH experiments were conducted to assess XTP6 expression. In brief, following dewaxing and rehydration, the samples were treated with 20 µg/ml proteinase K, fixed with 4% paraformaldehyde, and rinsed with distilled water. Subsequently, the samples were hybridized overnight at 42°C with double (5’ and 3’) digoxin-labeled XTP6 probe (BersinBio, China), and then incubated at 4 °C overnight with anti-digoxin monoclonal antibody conjugated to alkaline phosphatase. Finally, the samples were stained with nitro blue tetrazolium/5-bromo-4-chloro-3-indolylphosphate and observed under a microscope. As for IHC analysis, paraffin-embedded specimens underwent Ki67 staining.* Tissue sections were first incubated with normal goat serum for 30 min to block nonspecific binding, and then they were incubated with the primary antibody at 4 °C overnight. Subsequently, the avidin-biotin peroxidase system, complemented by DAB substrate, facilitated antigen localization. Nuclei counterstaining was performed using hematoxylin. To assess XTP6 expression in GBM tissues, the histochemical score (H-score) was employed. The calculation of an H-score for each specimen was performed by multiplying the staining intensity by the percentage of positively stained cells, resulting in scores between 0 and 300. These scores were subsequently utilized in statistical analyses. Samples were categorized based on expression levels as either low (score < 50) or high (score ≥ 50).

### RNA extraction and qRT-PCR analysis

RNA extraction from GBM tissues and cells was performed with the Simply P Total RNA Extraction Kit (Bioflux, China). Subsequently, the extracted RNA was reverse-transcribed into cDNA employing the MonScript RTIII All-in-One Mix with dsDNase (Monad, China). The qRT-PCR assay was conducted utilizing a MonAmp RapidStart Universal SYBR Green qPCR Mix (Monad, China). The sequences of primers can be found in Supplementary Table S1.

### Cell lines and cell culture

The GBM cell lines, namely T98G, A172MG, U87MG, LN229, U118MG, and U251MG, were acquired from the American Type Culture Collection (ATCC, USA). The maintenance of these GBM cell lines was carried out in Dulbecco’s Modified Eagle’s Medium (DMEM, Gibco, USA), supplemented with 10% fetal bovine serum (FBS, Gibco, USA) and a combination of antibiotics (Gibco, USA). Additionally, the normal human astrocyte (NHA) cell line, achieved from the Culture Collection of the Chinese Academy of Sciences, was maintained in basal astrocyte medium enriched with 2% FBS and 1% astrocyte growth supplement. *Primary cells, isolated from GBM tissues, were seeded into cell culture flasks pre-coated with poly-L-lysine. The flasks contained F-12/DMEM (Gibco, USA) supplemented with 2% 1×B27 (Gibco, USA), Recombinant Murine EGF (Peprotech, USA, 20 ng/ml), and Recombinant Murine FGF-basic (Peprotech, USA, 20 ng/ml).* Incubation conditions for the cell lines were established at 37 °C in an environment containing 5% CO_2_.

### FISH assay

We performed RNA fluorescence in situ hybridization (RNA-FISH) using an XTP6-specific probe (BersinBio, China). GBM cells were fixed using 4% paraformaldehyde for 15 min, and then incubated overnight with the probe at room temperature. Following fixation, the cells were blocked using 3% bovine serum albumin (BSA). Finally, DAPI staining was applied to the GBM cells, and images were acquired utilizing confocal laser scanning microscope (Leica, Germany).

### Nucleocytoplasmic fractionation assays

Trypsin-EDTA was used to detach adherent cells, which were then resuspended in PBS. Nuclear and cytoplasmic fractions were isolated from GBM cells using NE-PER™ Nuclear and Cytoplasmic Extraction Reagents (Thermo Scientific, USA), adhering to the guidelines provided by the manufacturer. In summary, for the process of fractionating plasma, approximately 10^7 cells were collected, followed by a PBS wash. Subsequently, we applied ice-cold CER I and CER II, allowing the mixture to incubate for 10 min at 0 °C. Subsequently, the mixture was centrifuged at 16,000×g for 5 min, with the supernatant being carefully preserved. The remaining pellet was resuspended in ice-cold NER, with vortexing intervals of 15 s every 10 min over a span of 40 min. Following centrifugation at 16,000×g for 10 min, the supernatant, designated as the nuclear fraction, was collected. Both nuclear and cytoplasmic fractions were then preserved at -80 °C for subsequent analyses.

### Plasmid construction, lentiviral packaging and cell transfection

Genechem Company (Shanghai, China) was responsible for the design and construction of all overexpression and knockdown plasmids utilized in this research. All siRNAs sequences were detailed in Supplementary Table S2. The study employed specific siRNAs, including XTP6 siRNA (si-XTP6), NDH2 siRNA (si-NDH2), c-myc siRNA (si-c-myc), and a scrambled siRNA (si-NC), all of which were sourced from Genechem Company (Shanghai, China). Transfection of these plasmids was carried out utilizing Lipofectamine 3000 (Invitrogen, USA), following the protocols specified by the manufacturer. In addition, to construct stably transduced cell lines, plasmids created by Genechem (Shanghai, China) were used for lentivirus packaging.

### CCK-8 assay

GBM cells were transfected with either siRNA or an overexpression plasmid. Afterward, the cells were seeded in 96-well plates at a density of 2 × 10^3 cells per well and incubated overnight. Following this, the cell viability was evaluated employing the Cell Counting Kit-8 (CCK-8) assay, as per the guidelines provided with the CCK-8 kit (Glpbio, USA). Viability measurements, indicated by OD450 absorbance, were conducted with a microplate reader (Thermo Fisher, USA) at 24-hour intervals over a span of four days.

### Colony formation assay

After transfection with either an overexpression plasmid or siRNA, GBM cells were cultured in 6-well plates (1000 cells/well). The cells were then incubated for a duration of two weeks. Subsequently, colonies were stained using 0.1% crystal violet to facilitate observation. The visible colonies were counted manually.

### EdU assay

GBM cells, post-transfection, were plated into 24-well plates (2 × 10^4^ cells/well) and incubated for three days. Following this incubation period, the cells underwent exposure to EdU reagent for two hours. Fixation was achieved using 4% paraformaldehyde and 0.5% Triton X-100. For nuclear staining, Hoechst stain was applied. The incorporation rate of EdU was quantified using ImageJ software.

### Wound healing assay

When the transfected GBM cell monolayers in 6-well plates achieved 85% confluency, a sterile 10 µl pipette tip was used to generate a wound by scratching. Photographs of the wound were captured at two distinct time intervals (0 and 24 h).

### Transwell assay

In the transwell invasion and migration assays, 5 × 10^4 cells were seeded into the upper chambers of 24-well plates (Corning, USA), following the instructions specified by the manufacturer. Following 48 h post-transfection, cells were obtained and resuspended in a serum-free medium. In the migration assay, these cells were then introduced into the upper chamber of uncoated transwell inserts. In the invasion assay, the membranes of the upper chambers were pre-coated with Matrigel (Yeasen, China) at a dilution of 1:8. To the lower chambers, 500 µL of medium enriched with 25% FBS was added. Following incubation for either 24–48 h at 37 °C, cells that had migrated or invaded were observed under a microscope.

### Neurosphere formation assay

*Transfected primary cells were plated in 24-well plates at a density of 300 cells per well and cultured for 7 days. Once neurospheres had formed, images were captured using a light microscope. The relative sizes of the neurospheres were then measured and calculated.*

### RNA pull-down assays and mass spectrometry anaysis

The RNA pull-down assays were performed with the BersinBio™ RNA Pull-down Kit (BersinBio, China), adhering strictly to the provided protocols. Each assay involved the utilization of a biotin-labeled RNA probe and protein extracts. Proteins that interacted with the biotin-labeled RNA probes were subsequently identified. For the identification of interacting proteins, mass spectrometry (MS) analyses were performed by LC-Bio Technologies (Hangzhou, China) on a blind basis, and for confirmation, the proteins underwent SDS-PAGE followed by western blotting analysis.

### Western blotting analysis

Total protein was isolated using radioimmunoprecipitation assay buffer (RIPA) (Solarbio, China) supplemented with protease inhibitors. The proteins were subsequently separated on a 10% SDS-PAGE gel and transferred onto a PVDF membrane. The membrane was blocked by incubating it in 5% skim milk at room temperature for 1 h. The membranes were incubated with the primary antibodies overnight at 4 °C, and then with secondary antibodies. The antibodies and agents are detailed in Supplementary Table S3. Protein detection was performed by utilizing the Chemiluminescent Imaging System (Tanon, China).

### RIP assays

RIP assays were performed with the BersinBio™ RIP Kit (BersinBio, China), following the provided protocol. Approximately 2 × 10^7^ U118MG or U251MG cells were lysed using the kit’s RIP lysis buffer. Lysates were then incubated with either specific antibodies or control IgG from mouse or rabbit, conjugated to magnetic beads. RNA isolated from these complexes underwent qRT-PCR for analysis. Normal IgG was regarded as the negative control, whereas GAPDH served as the non-specific control.

### ChIRP assays

ChIRP assays were performed with the BersinBio™ Chromatin Isolation by RNA Purification Kit (BersinBio, China), following the guidelines provided by the manufacturer. Biotin probes targeting XTP6 were designed via an online single-molecule FISH designer and categorized into odd and even sets (Supplementary Table S4). For each ChIRP experiment, 2 × 10^7 GBM cells were prepared and crosslinked to facilitate each hybridization reaction. The cell lysates were sonicated to obtain DNA fragments, followed by hybridization with the probes for 4 h at 37 °C. The DNA and RNA were subsequently extracted and purified from the hybridized beads for analysis via qRT-PCR.

### ChIP assays

ChIP assays were conducted with the Chromatin Immunoprecipitation Kit (CST, USA). Stable transfected cells underwent fixation with 1% formaldehyde for 10 min at room temperature, followed by lysis using the provided Sonication Cell Lysis Buffer from the kit. DNA was sheared into fragments ranging from 100 to 400 nucleotides through ultrasonication. Antibodies against c-myc and H3K27me3 were used in the assays. The immunoprecipitated DNA was then evaluated by qRT-PCR analysis. The primer sequences were displayed in Supplementary Table S5.

### Dual-luciferase reporter assays

Specific segments of the IκBα promoter were straightly cloned into the pGL4 luciferase reporter vector. GBM cells were co-transfected with pcDNA4.1-c-myc and pcDNA4.1-XTP6. The pGL4 vector served as a negative control. Luciferase activity was evaluated by performing the Dual-Luciferase Reporter Assay System (Promega, USA), with Renilla luciferase activity normalized to Firefly luciferase activity to assess transfection efficiency within each experiment.

#### Bioinformatics analysis

The promoter of XTP6 was identified through the UCSC database (http://www.genome.ucsc.edu/). Prediction of c-myc binding sites within the XTP6 promoter utilized the JASPAR database (http://jaspardev.genereg.net/). Additionally, the underlying binding sites of XTP6 and IκBα promoter were predicted on the website (http://www.gaemons.net/LongTarget).

### Construction of intracranial xenograft mouse model

The ethical approval for animal experiments conducted in this research was granted by the Animal Experiment Ethics Committee of Nanchang University (Approval No. NCULAE-20,221,031,035). *Male* BALB/c nude mice, aged 5 weeks, were utilized to establish intracranial xenograft models of GBM (GemPharmatech, China). Cells from the U251MG line, either with overexpression or knockdown of XTP6 and their respective negative controls, were prepared in pre-chilled PBS. For the inoculation, 3 × 10^5^ U251MG cells in 6 µL PBS were injected into the right frontal lobe of the mice under anesthesia with isoflurane. The precise inoculation site was determined to be 2 mm lateral and 1 mm posterior to the anterior fontanelle. Tumor growth was monitored via the intensity of luciferase expression, measured using the IVIS Lumina Series III system (PerkinElmer, USA). Mice exhibiting abnormal behaviors or seizures were humanely euthanized by cervical dislocation. In treatments targeting GBM, either PBS or JSH was implemented intravenously at a dosage of 10 mg/kg. Eventually, brains were excised and preserved in 4% paraformaldehyde for subsequent histological analysis, including hematoxylin-eosin (HE) staining and IHC evaluations.

### Statistical analysis

In the survival analysis, a two-sided log-rank test was employed to assess differences in prognosis between low-XTP6 and high-XTP6 subgroups of GBM. For two groups comparisons, two-tailed Student’s t-tests were employed. Additionally, for analyses involving multiple groups, a two-way ANOVA with Tukey’s tests was conducted. The statistical analyses were conducted utilizing R software, version 4.1.0, GraphPad Prism, version 8, and ImageJ. P-values below 0.05 were deemed statistically significant.

## Results

### Upregulated XTP6 expression was connected with adverse prognosis in GBM

**Fig. 1:**
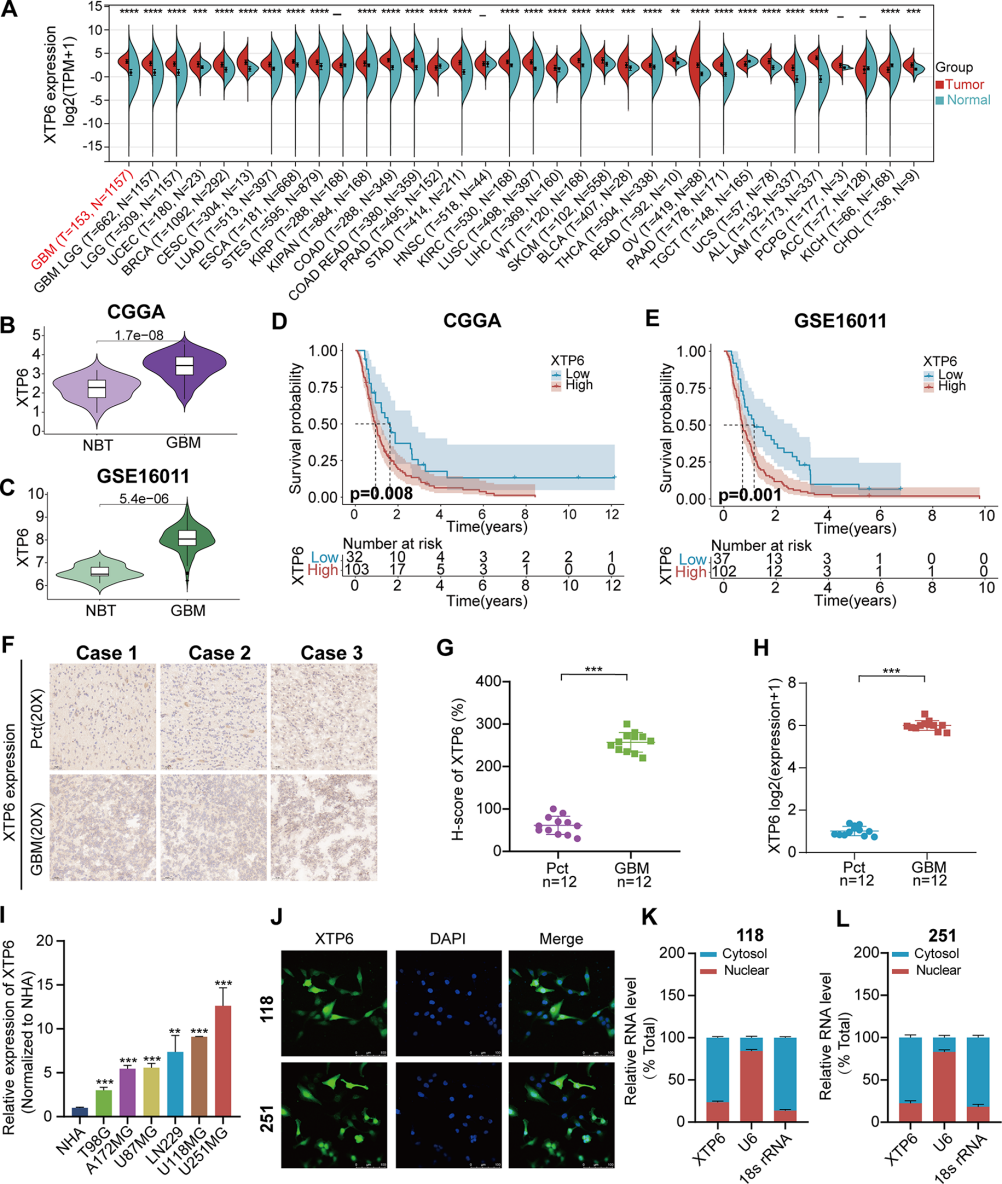
Overexpression of XTP6 is connected with adverse prognostic outcomes in GBM (**A**) The differential expression of XTP6 in diverse tumor tissues and their respective normal tissues. (**B**-**C**) The differential expression of XTP6 in CGGA (**B**) and GSE16011 (**C**) GBM datasets. (**D**-**E**) Prognostic analysis of the low-XTP6 and high-XTP6 expression subgroups in CGGA (**D**) and GSE16011 (**E**) GBM datasets. The cutoff value represented the median expression of XTP6. (**F**-**G**) *RNA-ISH* analysis evaluated the expression of XTP6 in the GBM tissues and corresponding PCTs (**F**). H-score of XTP6 between the GBM tissues and corresponding PCTs (**G**). (**H**) qRT-PCR analysis of XTP6 expression in GBM tissues and corresponding PCTs. (**I**) qRT-PCR analysis of XTP6 expression in GBM and NHA cell lines. (**J**) FISH analysis indicated the subcellular distribution of XTP6 in U118MG and U251MG cells. (**K**-**L**) Subcellular fractionation assays verified the subcellular distribution of XTP6 in U118MG and U251MG cells. (*P < 0.05, **P < 0.01, ***P < 0.001)

First, we performed pan-cancer analysis of XTP6 expression. Significant disparities in the expression of XTP6 between diverse cancer types and normal tissues were observed. The XTP6 expression level was markedly higher in GBM than in corresponding normal tissues (Fig. 1A). Subsequently, we conducted additional investigations into the expression of XTP6 in two independent GBM datasets. Our findings indicated that the expression levels of XTP6 in GBM were obviously higher compared to those in normal brain tissues (NBT) in both CGGA (Fig. 1B) and GSE16011 datasets (Fig. 1C). In addition, the results of survival analysis revealed that the prognosis of GBM patients in high-XTP6 expression subgroup was markedly poorer than in low-XTP6 expression subgroup in both CGGA (Fig. 1D) and GSE16011 (Fig. 1E) datasets.

Subsequently, we conducted *RNA-ISH* analysis on 12 cases of GBM tissues and corresponding PCTs, revealing a distinctly higher expression level of XTP6 in GBM tissues compared to PCTs. (Fig. 1F, G). Moreover, through qRT-PCR experimental analysis, we observed a markedly higher expression level of XTP6 in GBM tissues compared to PCTs (Fig. 1H).

Additionally, we inspected the expression of XTP6 in six GBM cell lines (T98G, A172MG, U87MG, LN229, U118MG, and U251MG) and a NHA cell line. The results indicated that XTP6 expression was significantly higher in GBM cell lines than in NHA cell line and the highest expression of XTP6 was found in both U251MG and U118MG (Fig. 1I). Therefore, U251MG and U118MG cells were selected for further study. Subsequently, the cellular distribution of XTP6 within U251MG and U118MG cells was determined through FISH and subcellular fractionation analyses. Our *findings* revealed that XTP6 was present in both nuclear and cytoplasmic in U251MG and U118MG cells (Fig. 1J-L). In summary, XTP6 is a significant oncogene and is connected with adverse prognosis of GBM.

### XTP6 facilities malignant progression of GBM cells

**Fig. 2:**
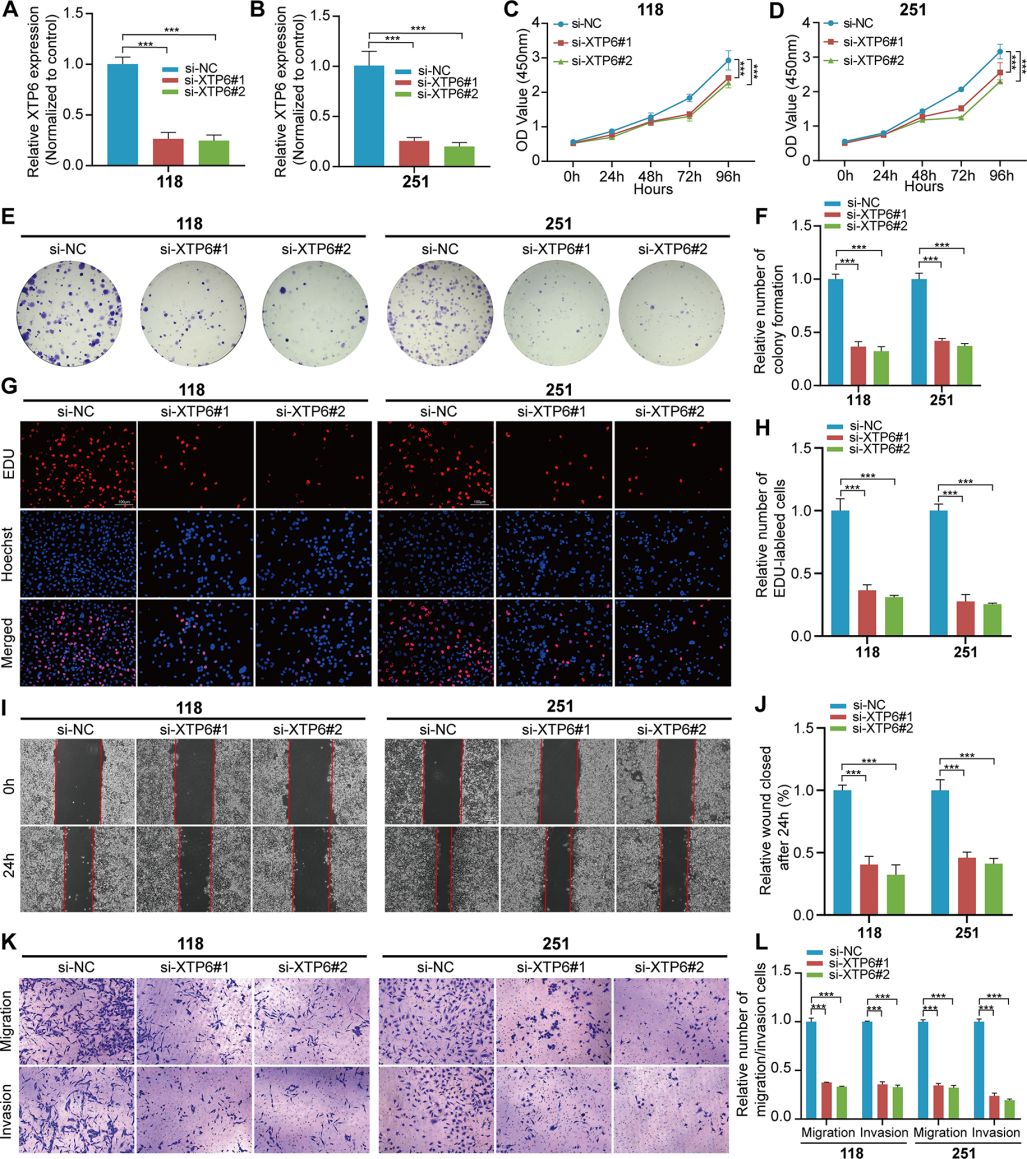
Knockdown of XTP6 inhibits proliferation, migration, and invasion of GBM cells. (**A** and **B**) qRT-PCR assays confirmed the efficiencies of XTP6 knockdown in U118MG (**A**) and U251MG (**B**) cells. (**C**-**D**) The cell viability of si-XTP6-transfected U118MG (**C**) and U251MG (**D**) cells by CCK-8 assays. (**E**-**F**) Effect of XTP6 knockdown on colony formation was counted in U118MG and U251MG cells (**E**). Histogram analysis revealed the mean ± standard deviation (SD) of colony counts across three independent experiments (**F**). (**G**-**H**) Representative images (**G**) and histogram analysis (**H**) displayed the outcomes of EdU assays following the knockdown of XTP6 in U118MG and U251MG cells. (**I**-**J**) Representative images (**I**) and histogram analysis (**J**) illustrated the results of wound healing assays following XTP6 silencing in U118MG and U251MG cells. (**K**-**L**) Representative images (**K**) and histogram analysis (**L**) depicted the effects of XTP6 knockdown on Transwell assays in U118MG and U251MG cells. (*P < 0.05, **P < 0.01, ***P < 0.001)

To determine whether XTP6 contributed to GBM malignant progression, we performed cells functional experiments. In U118MG and U251MG cells, we manipulated the expression levels of XTP6 by employing specific plasmids: one set for knockdown and another for overexpression of XTP6. This approach allowed us to effectively decrease or increase XTP6 expression, respectively (Fig. 2A, B and Fig. S1A). The CCK-8 assays disclosed an obvious decrease in the viability of U118MG and U251MG cells upon downregulation of XTP6 (Fig. 2C, D), while the overexpression of XTP6 led to increased cell viability in both U118MG and U251MG cells (Fig. S1B, C). Colony formation assays demonstrated a significant decrease in cell colonies following the knockdown of XTP6, in contrast to the NC (Fig. 2E, F), while the overexpression of XTP6 exhibited an inverse effect (Fig. S1D, E). Furthermore, suppressing XTP6 expression substantially hindered cell proliferation, as evidenced by EdU assays in U118MG and U251MG cell lines (Fig. 2G, H). Conversely, enhancing XTP6 expression notably facilitated proliferation within these same cell lines (Fig. S1F, G). The results indicate that XTP6 is pivotal for the proliferation of GBM cells in vitro.

Additionally, our research verified that the upregulation of XTP6 enhances the migration and invasion capabilities of GBM cells. Through wound healing assays, we observed that reducing XTP6 expression significantly hindered the mobility of GBM cells (Fig. 2I, J), while its upregulation produced a contrary outcome (Fig. S1H, I). Through transwell assays, we confirmed that the migration and invasion abilities of U118MG and U251MG cells were weakened after silencing XTP6 (Fig. 2K, L), the opposite results were observed after overexpressing XTP6 (Fig. S1J, K). Together, our *findings* demonstrated that upregulation of XTP6 facilities the migration and invasion of GBM cells in vitro.

### XTP6 promotes GBM initiation in vivo

To investigate whether knocking down XTP6 could impact the *initiation* of GBM in vivo, we utilized the immunodeficient nude mice as an in vivo model (Fig. S2A). The results demonstrated that nude mice in the sh-XTP6#1 and sh-XTP6#2 groups showed an apparent reduction in the volume of intracranial tumors (Fig. S2B, C), exhibited weaker overall fluorescence intensity (Fig. S2D), experienced a slower decline in body weight (Fig. S2E), and had longer overall survival times (Fig. S2F) when compared to sh-NC group. Additionally, IHC analyses were conducted on the tumor tissues removed from the nude mice. These analyses externalized that the percentage of Ki67-positive cells within the tumor samples from the sh-XTP6#1 and sh-XTP6#2 groups was obviously reduced compared to that in the tumor samples from the sh-NC group (Fig. S2G, H). Therefore, reducing the expression of XTP6 can inhibit the *initiation* of GBM in vivo.

### XTP6 straightly interacts with NDH2

**Fig. 3:**
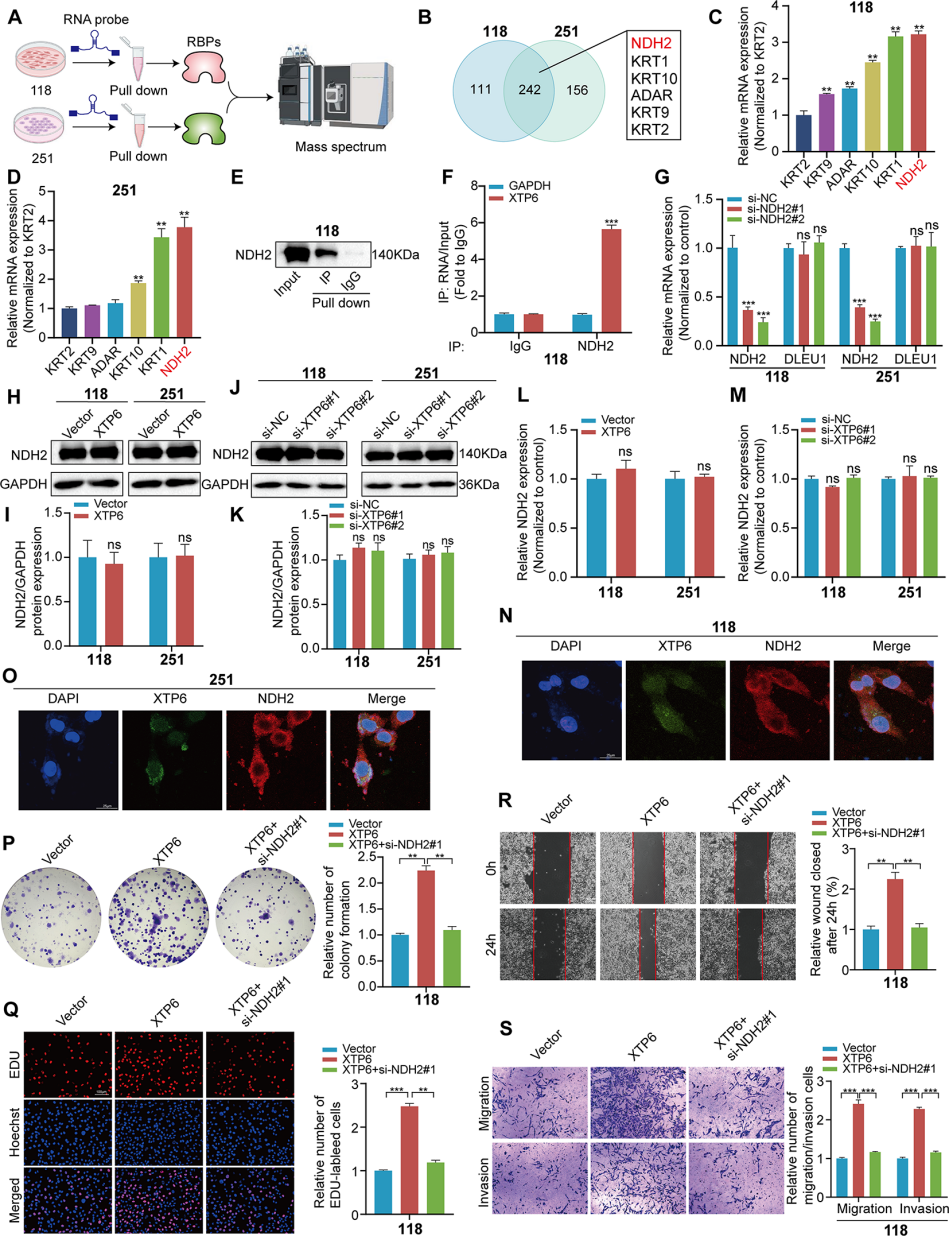
XTP6 directly binds to NDH2. (**A**) RNA pull-down assays were conducted in U118MG and U251MG cells, followed by mass spectrometry for analysis and identification. (**B**) The results of mass spectrometry suggested a probable interaction between XTP6 and NDH2. (**C**-**D**) qRT-PCR analysis revealed that NDH2 exhibited the highest expression levels at the mRNA stage in U118MG (**C**) and U251MG (**D**) cells. (**E**) Western blotting analysis of proteins obtained by XTP6 probes, suggesting that XTP6 interacts with NDH2 in U118MG cells. (**F**) RIP assays indicated that XTP6 bound to NDH2 in U118MG cells. (**G**) qRT-PCR analysis demonstrated the effectiveness of NDH2 knockdown and the expression levels of XTP6 in U118MG and U251MG cells with NDH2 suppression. (**H**-**K**) Western blotting analysis revealed the NDH2 expression after XTP6 overexpression (**H, I**) or knockdown (**J, K**) in U118MG and U251MG cells. (**L**-**M**) qRT-PCR analysis indicated the NDH2 expression after XTP6 overexpression (**L**) or knockdown (**M**) in U118MG and U251MG cells. (**N**, **O**) The colocalization of XTP6 and NDH2 was assessed by FISH and immunofluorescence in U118MG (**N**) and U251MG cells (**O**). (**P-S**) Colony formation (**P**), EdU (**Q**), Wound healing (**R**), and Transwell (**S**) assays demonstrated that knockdown of NDH2 partly reversed the impact of XTP6 overexpression in U118MG cells. (*P < 0.05, **P < 0.01, ***P < 0.001)

LncRNAs have the capacity to modulate biological functions through their interaction with proteins, and they play a pivotal role in facilitating malignant progression in cancer by mediating a range of signaling pathways [13, 27]. Therefore, we executed RNA pull-down experiments to detect proteins that interact with XTP6 by employing biotinylated XTP6 probe in U118MG and U251MG cell lines, followed by protein identification through mass spectrometry (MS) analysis (Fig. 3A). The findings indicated that a total of 242 proteins were commonly identified in the RNA pull-down assays performed with U118MG and U251MG cells. From these, six proteins (NDH2, KRT1, KRT10, ADAR, KRT9, and KRT2) were selected based on their high abundance in the pull-down samples, with NDH2 being the most prevalent (Fig. 3B). Following this, the transcriptional expression of these six genes was further confirmed via qRT-PCR assays in U118MG and U251MG cells, revealing that NDH2 exhibited the highest mRNA expression levels (Fig. 3C, D). Additionally, western blotting analysis corroborated the association between XTP6 and NDH2 in U118MG and U251MG cells, as initially suggested by the RNA pull-down assays (Fig. 3E and S3A). To ascertain the direct interaction between NDH2 and XTP6, we conducted RIP assays. The results revealed a significant association between XTP6 and NDH2 in the U118MG and U251MG cells (Fig. 3F and S3B).

Nevertheless, the reduction of NDH2 expression levels did not transform the expression of XTP6 (Fig. 3G), and similarly, the modulation of XTP6, whether by overexpression or knockdown, had no impact on the protein (Fig. 3H-K) and mRNA (Fig. 3L, M) expression levels of NDH2. *Additionally, FISH and immunostaining revealed the co-localization of XTP6 and NDH2 in U118MG and U251MG cells (*Fig. 3N-O*).* The above results suggested that XTP6 was capable of directly interacting with NDH2; however, there appeared to be no reciprocal regulatory relationship between them.

Considering the contribution of NDH2 to the progression of various cancers, we delved further into its role as a potential oncogene in GBM. First, we detected that NDH2 expression levels were notably elevated in GBM tissues compared to NBT in TCGA dataset (Fig. S3C). To validate the expression of NDH2 in GBM, western blotting and qRT-PCR analyses were performed on samples from 12 GBM cases and their corresponding PCTs. The outcomes demonstrated that both mRNA (Fig. S3D) and protein (Fig. S3E, F) expression levels of NDH2 in GBM tissues were distinctly higher compared to those in PCTs. The efficacy of NDH2 knockdown was confirmed via western blotting assays in U118MG and U251MG cells, revealing a significant reduction in NDH2 expression (Fig. S3G-I).

Subsequently, rescue experiments were conducted to investigate whether the correlation between NDH2 and XTP6 contributed to GBM progression. CCK-8 assays verified that overexpressing XTP6 enhanced the viability of U118MG and U251MG cells, while the suppression of NDH2 partially mitigated these effects (Fig. S3J, K). Colony formation assays demonstrated a notable enhancement in cell colony numbers upon XTP6 overexpression in U118MG and U251MG cells, whereas NDH2 knockdown partially counteracted these outcomes (Fig. 3P and Fig. S3L). Furthermore, EdU assays suggested that the upregulation of XTP6 expression can facilitate cell proliferation, with the silencing of NDH2 partially reversing these effects (Fig. 3Q and S3M). Our *findings* indicate that the suppression of NDH2 expression can counteract the proliferative influence induced by elevated levels of XTP6 in GBM cells.

Furthermore, wound healing assays displayed that the upregulation of XTP6 markedly enhanced the motility of GBM cells, whereas silencing NDH2 expression partially negated these enhancements (Fig. 3R and S3N). The transwell assays revealed an increase in the migratory and invasive capabilities of GBM cells following the upregulation of XTP6, whereas the reduction of NDH2 expression partially attenuated these phenomena (Fig. 3S and S3O). The findings demonstrate that the suppression of NDH2 expression can partially counteract the enhanced migratory and invasive properties induced by the overexpression of XTP6 in GBM cells.

### XTP6 activates the NF-κB signaling pathway by regulating IκBα expression

**Fig. 4:**
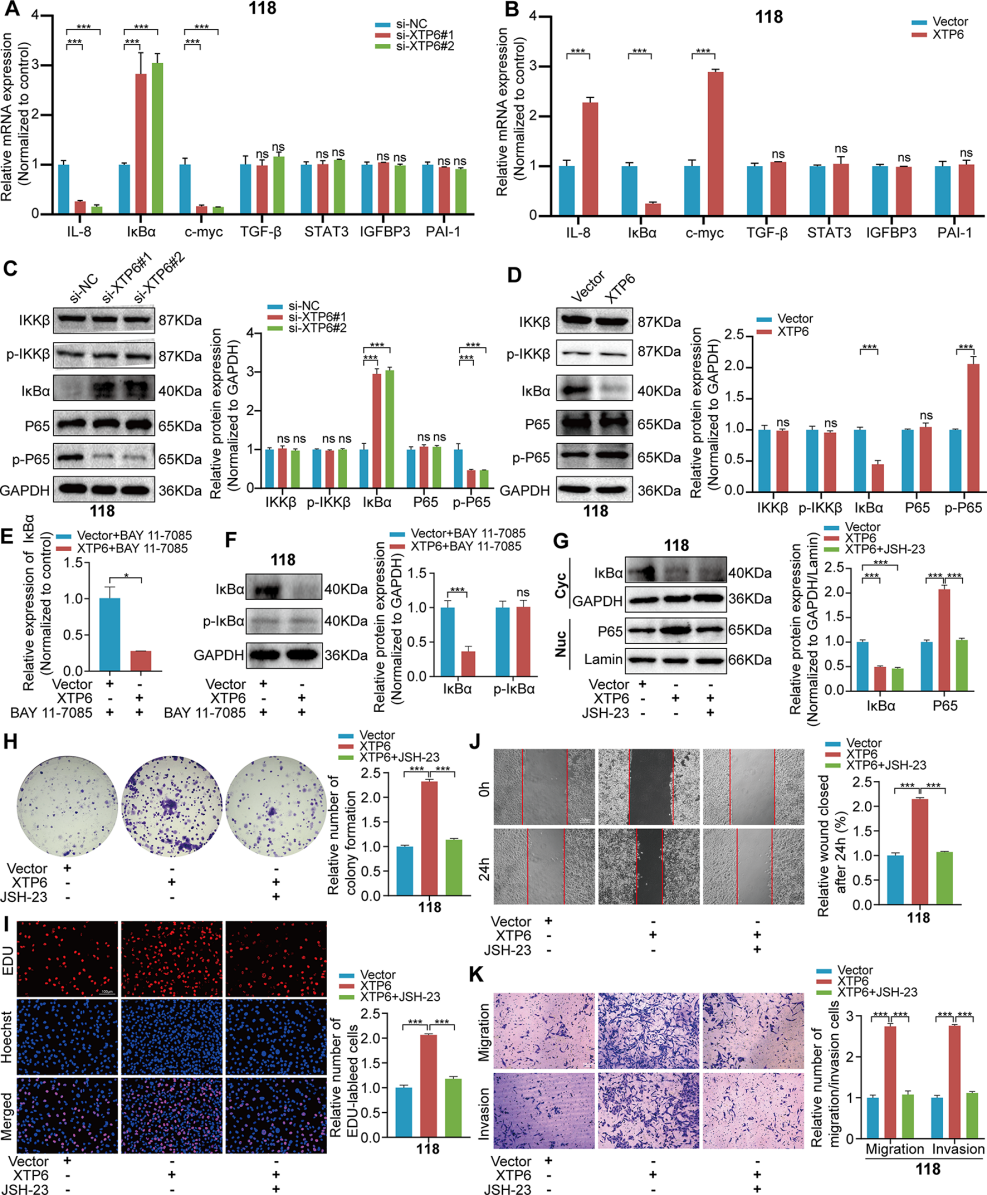
XTP6 activates the NF-κB signaling pathway through downregulating the IκBα expression in U118MG cells. (**A**-**B**) The expression of genes within the NF-κB signaling pathway was assessed via qRT-PCR assay in U118MG cells subjected to knockdown (**A**) or overexpression (**B**) of XTP6. (**C**-**D**) Western blotting analysis revealed alterations in protein levels associated with the NF-κB signaling pathway following the knockdown (**C**) or overexpression (**D**) of XTP6 in U118MG cells. (**E**-**F**) qRT-PCR and Western blotting analyses demonstrated that treatment with BAY 11–7085 led to a downregulation of IκBα at both mRNA (**E**) and protein (**F**) levels in U118MG cells mediated by XTP6. (**G**) Western blotting assays suggested that JSH-23 can reverse the translocation of P65 mediated by XTP6 in U118MG cells. (**H-K**) Colony formation (**H**), EdU (**I**), Wound healing (**J**), and Transwell (**K**) assays indicated that JSH-23 can reverse the effects of XTP6-overexpressing U118MG cells. (*P < 0.05, **P < 0.01, ***P < 0.001)

NDH2, also known as DExH-Box helicase 9, is an RNA helicase. Previous research has elaborated that NDH2 plays a crucial part in activating the NF-κB signaling pathway [28]. Hence, we hypothesize that NDH2 may activate the NF-κB signaling pathway by functioning as an RNA helicase. Subsequently, we conducted qRT-PCR and western blotting analyses to evaluate alterations in genes associated with the NF-κB signaling pathway in GBM cells. *The outcomes demonstrated that the knockdown of XTP6 caused a rise in IκBα expression, whereas overexpression of XTP6 led to reduced IκBα expression in U118MG, U251MG and primary cells (*Fig. 4A-D, S4A-D and S5A-D). Nonetheless, alterations in XTP6 expression, either through overexpression or suppression, did not alter IKK phosphorylation levels in U118MG, U251MG and primary cells (Fig. 4C-D, S4C-D and S5C-D). The results suggested that XTP6 exerted its regulatory effects on the NF-κB signaling pathway by modulating IκBα expression, rather than through activating the IKK.

Prior research has indicated that the degradation of IκBα can result from either its phosphorylation or a reduction in IκBα transcription levels [29]. In this study, we introduced BAY 11-7085, known as an inhibitor of IκBα phosphorylation, to both GBM cells overexpressing XTP6 and NC cells to evaluate IκBα expression levels. *The outcomes showed that in U118MG, U251MG and primary cells, IκBα expression levels were lower in those treated with XTP6-overexpressing plasmids than in cells receiving the matching empty vectors after BAY 11-7085 application, implying that the regulation of IκBα expression by XTP6 mainly involves transcriptional control (*Fig. 4E-F, S4E-F and S5E-F).

Additionally, we investigated whether XTP6 influenced GBM progression by activating the NF-κB signaling pathway. JSH-23 is a small molecule inhibitor of NF-κB signaling that prevents the nuclear translocation of the NF-κB p65 subunit, thereby blocking the transcription of NF-κB target genes involved in cell proliferation. Our findings revealed that the upregulation of XTP6 expression intensified the activation of the NF-κB signaling pathway, and the application of the NF-κB inhibitor, JSH-23, markedly inhibited the activation phenomenon triggered by XTP6 (Fig. 4G, S4G and S5G). Furthermore, suppressing the NF-κB signaling pathway using JSH-23 partially reversed the malignant progression of GBM cells induced by XTP6 overexpression (Fig. 4H-K and S4H-K). Interestingly, the results demonstrated a decrease in the relative sizes of neurospheres in primary cells after XTP6 knockdown (Fig.S5H). Additionally, inhibiting the NF-κB signaling pathway with JSH-23 partially restored the relative sizes of neurospheres in primary cells affected by XTP6 overexpression (Fig.S5I). In conclusion, the results demonstrate that XTP6 can activate the NF-κB signaling pathway by reducing the expression level of IκBα transcript and thereby promotes the malignant progression of GBM.

### XTP6 interacts with the promoter regions of IκBα by forming triplex structures

**Fig. 5:**
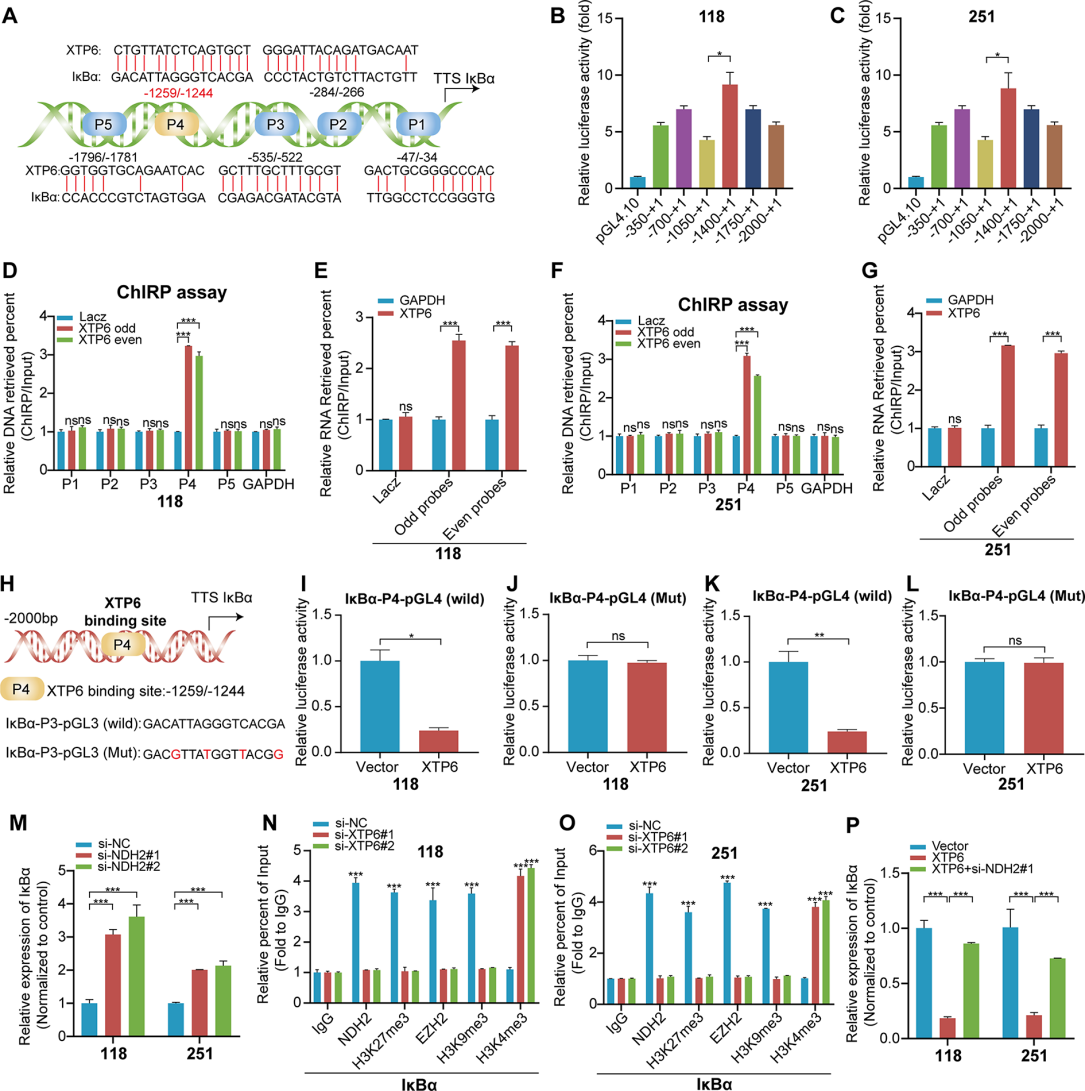
XTP6 interacts with the promoter region of IκBα to form triplex structures, leading to the downregulation of IκBα expression. (**A**) Illustrations depicting the putative binding sites of XTP6 within the promoter region of IκBα. (**B**-**C**) Luciferase reporter assays combined with progressive deletions were employed to investigate the transcriptional activity within the IκBα promoter region in U118MG (**B**) and U251MG (**C**) cells. (**D-G**) ChIRP analysis was conducted to examine chromatin associated with XTP6 in U118MG (**D, E**) and U251MG (**F, G**) cells. The isolated chromatin and RNA were then evaluated using qRT-PCR. (**H**) The IκBα promoter, featuring mutated XTP6 binding sites, alongside the wild-type IκBα promoter, were cloned into the pGL4-luciferase reporter vector. (**I-L**) Dual-Luciferase reporter assays were utilized to investigate the transcriptional activity of the IκBα promoter, comparing versions with wild-type and mutated XTP6 binding sites in U118MG (**I, J**) and U251MG (**K, L**) cells. (**M**) qRT-PCR analysis was employed to assess IκBα expression levels in the control and NDH2-silenced GBM cells. (**N**-**O**) ChIP-qPCR analysis was utilized to evaluate the NDH2, H3K27me3 status, and EZH2 occupancy within the IκBα promoter region after XTP6 knockdown in U118MG (**N**) and U251MG (**O**) cells. (**P**) qRT-PCR analysis demonstrated that knockdown of NDH2 reversed the downregulation of IκBα mediated by XTP6 in GBM cells. (*P < 0.05, **P < 0.01, ***P < 0.001)

To elucidate the molecular processes by which XTP6 affects the expression of IκBα, the possible binding sites of XTP6 and IκBα promoter were predicted through the bioinformatics analysis (Fig. 5A). Subsequently, we designed a suite of plasmids incorporating various truncations of the IκBα promoter, spanning from − 2000 nt to + 1 nt and these constructs were then evaluated through luciferase reporter assays. The results indicated an obvious reduction in luciferase activity following the transfection of plasmids carrying fragments from − 1400 to -1050 bp (Fig. 5B, C).

Additionally, the direct interaction between XTP6 and the IκBα promoter region was confirmed through ChIRP assays. The outcomes demonstrated binding of XTP6 to the IκBα promoter region between − 1259 and − 1244 bp in U118MG (Fig. 5D, E) and U251MG (Fig. 5F, G) cells, implying the development of a triplex configuration between XTP6 and the IκBα promoter. Collectively, our findings indicate that XTP6 attenuates IκBα transcription via the formation of a DNA-RNA triplex with sequences in the IκBα promoter.

### XTP6 facilitates the trimethylation of H3K27 at the IκBα promoter through its interaction with NDH2

To determine the impact of XTP6 on IκBα transcriptional activity, we constructed a pGL4 vector containing mutations in the IκBα promoter (Fig. 5H). Luciferase assay findings revealed that, following co-transfection with XTP6, the IκBα promoter exhibited markedly higher luciferase activity in U118MG (Fig. 5I, J) and U251MG (Fig. 5K, L) cells transfected with the mutant IκBα-pGL4 vector compared to those transfected with the wild-type IκBα-pGL4 vector. Previous studies have demonstrated that EZH2, as the enzymatic core of the Polycomb Repressive Complex 2 (PRC2), is essential in facilitating the trimethylation at lysine 27 of histone H3 (H3K27me3), thereby effectuating transcriptional silencing [30–32]. The NDH2 is known for its involvement in various molecular processes, including RNA processing and chromatin remodeling, which could potentially interact with mechanisms controlling histone modifications like H3K27me3 methylation. Therefore, we inspected whether NDH2 played a role in mediating the H3K27me3 on the promoter of IκBα. Firstly, our findings revealed an upregulation of IκBα expression in GBM cells subjected to NDH2 silencing (Fig. 5M). Furthermore, ChIP assays demonstrated that elevated levels of H3K27me3 and EZH2 were specifically enriched at the XTP6 binding site within the IκBα promoter, a process facilitated by the interaction with NDH2 (Fig. 5N, O). *Similarly, our ChIP experiments showed that elevated levels of H3K9me3, a marker associated with gene silencing, were also enriched at the same XTP6 binding site within the IκBα promoter, indicating a similar regulatory mechanism involving NDH2 (*Fig. 5N, O*)*. Additionally, the suppression of NDH2 expression was found to reverse the reduction in IκBα expression caused by XTP6 (Fig. 5P). Collectively, the results suggest that XTP6 mediates the downregulation of IκBα expression via H3K27me3 methylation in an NDH2 dependent manner.

### XTP6 maintains the activation of the NF-κB signaling pathway by establishing a positive feedback loop with c-myc

**Fig. 6:**
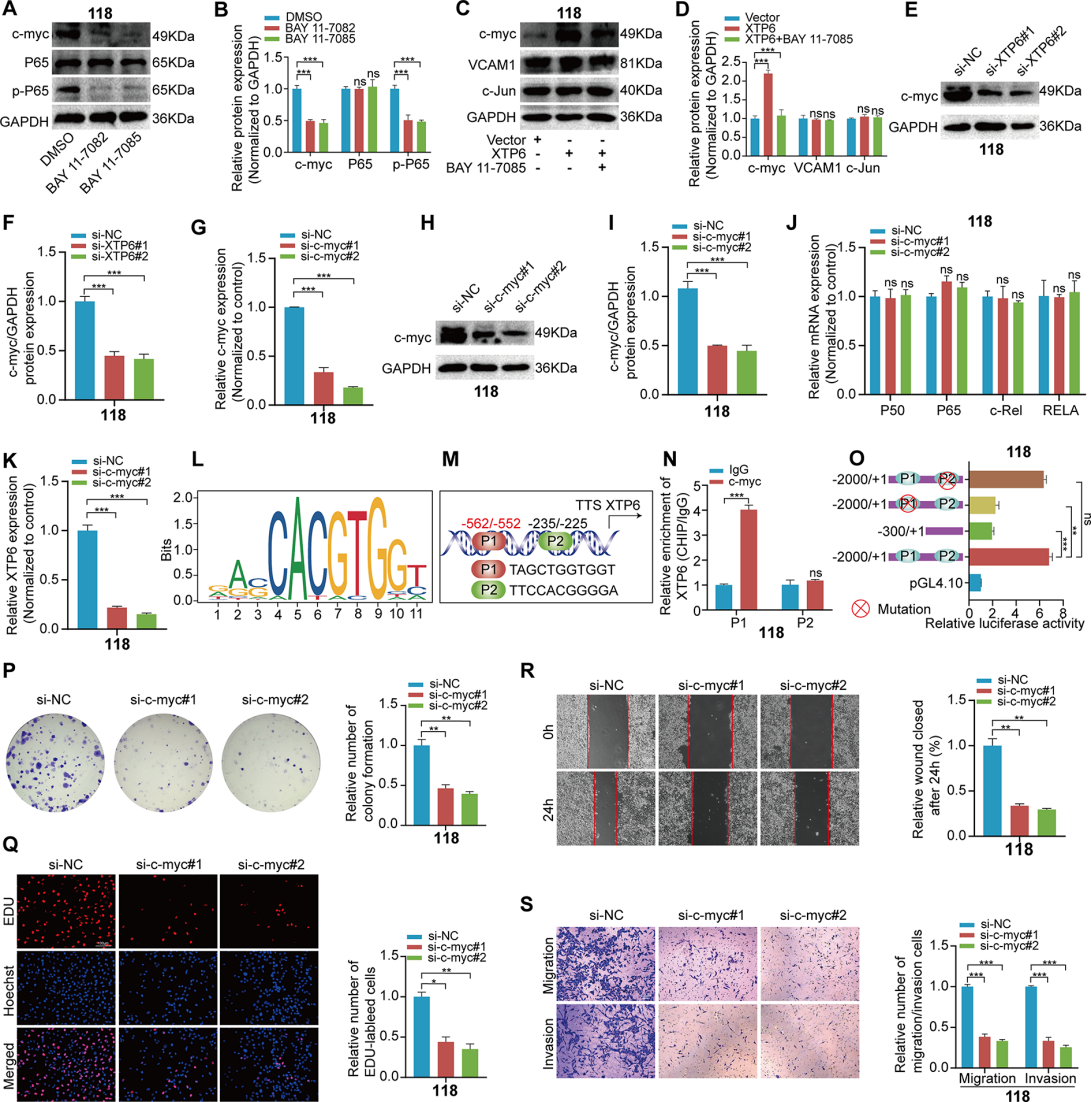
XTP6 promotes sustained activation of the NF-κB signaling pathway by establishing a positive feedback loop with c-myc in U118MG cells. (**A**-**B**) Western blotting analysis showed that administering NF-κB inhibitors, specifically BAY 11-7082 and BAY 11-7085, led to a reduction in the expression levels of c-myc and p-p65 in U118MG cells. (**C**-**D**) Western blotting analysis indicated that after treating XTP6-overexpressing U118MG cells with BAY 11-7085, the expression levels of c-myc, VCAM1, and C-Jun were assessed 72 h post-treatment. (**E**-**F**) Western blotting analysis was employed to assess the expression of c-myc after XTP6 knockdown in U118MG cells. (**G-I**) qRT-PCR **(G)** and Western blotting (**H, I**) analyses were utilized to inspect the efficiencies of c-myc knockdown in U118MG cells. **J** qRT-PCR assays revealed that the depletion of c-myc did not affect the expression levels of P50, P65, c-Rel, and RELA in U118MG cells. (**K**) qRT-PCR analysis demonstrated that c-myc depletion led to a decrease in XTP6 expression in U118MG cells. (**L**) Schematic diagram of the binding motif of the transcription factor c-myc. (**M**) A schematic representation was developed to illustrate the predicted c-myc binding sequences within the promoter region of XTP6. (**N**) ChIP-qPCR analysis was performed in U118MG cells. (**O**) Luciferase reporter assays demonstrated that the depletion of P1 can lead to a reduction in the transcriptional activity of the XTP6 promoter in U118MG cells. (**P-S**) Colony formation (**P**), EdU (**Q**), Wound healing (**R**), and Transwell (**S**) assays showed that knockdown of c-myc can inhibit the proliferation, migration and invasion of U118MG cells. (*P < 0.05, **P < 0.01, ***P < 0.001)

In line with expectations, XTP6 contributed to the activation of the NF-κB signaling pathway by diminishing IκBα expression. However, the downstream regulatory factors of NF-κB signaling pathway that are connected with GBM progression remained unidentified. Numerous studies have demonstrated that transcription factor c-myc is a crucial downstream factor of the NF-κB signaling pathway [33–36]. Interestingly, western blotting assays demonstrated an apparent reduction in c-myc expression following the application of NF-κB signaling pathway inhibitors (Fig. 6A, B and S6A, B). *Additionally, we investigated the changes in c-myc expression within GBM cells that were engineered to overexpress XTP6 were treated with BAY 11-7085*. The findings disclosed that blocking the NF-κB signaling pathway markedly reduced c-myc expression in comparison to cells that were artificially overexpressing XTP6 (Fig. 6C, D and S6C, D). Similarly, western blotting analyses exhibited that the silencing of XTP6 substantially lowered the protein expression of c-myc (Fig. 6E, F and S6E, F). Subsequently, we modulated c-myc expression levels using knockdown plasmids targeted at c-myc, effectively reducing its expression (Fig. 6G-I and S6G-I). Additionally, we detected that the silencing of c-myc did not influence the expression levels of P50, P65, c-Rel, and RELA (Fig. 6J and S6J). These *findings* suggest that c-myc represents a critical downstream factor of the NF-κB signaling pathway in GBM cells.

The formation of a positive feedback loop played a crucial role in the malignant progression of cancer [37, 38]. Moreover, we conducted further analysis to detect the impact of alterations in c-myc expression on the transcriptional expression levels of XTP6. The outcomes verified that the suppression of c-myc led to a decrease in XTP6 expression, whereas the upregulation of c-myc enhanced XTP6 expression in GBM cells (Fig. 6K and S6K, L). Furthermore, bioinformatics analysis of the XTP6 promoter identified two underlying c-myc binding sites, referred to as P1 and P2 (Fig. 6L, M).

To verify the correlation between c-myc and the predicted site on the XTP6 promoter, ChIP assays were conducted, demonstrating that c-myc could directly interact with the P1 site of the XTP6 promoter (-562 bp to-552 bp) (Fig. 6N and S6M). Additionally, the outcomes of luciferase assay outcomes indicated that the luciferase expression driven by c-myc was significantly reduced by mutation at the P1 site, whereas mutations at P2 had no discernible impact (Fig. 6O and S6N). This implies that the transcription factor c-myc interacts with the XTP6 promoter specifically through the P1 site in GBM cells. *Furthermore, our research revealed that reducing c-myc expression led to a suppression of malignant progression in GBM cells, likely due to the direct negative impact of c-myc inactivation on the oncogenic pathways driving GBM* (Fig. 6P-S and S6O-T).

In short, these results indicate that lncRNA-XTP6 promotes the activation of the NF-κB signaling pathway by creating a positive feedback loop with c-myc, which facilitates the malignant advancement of GBM.

### Inhibition of NF-κB signaling pathway reverses XTP6 mediated GBM progression in vivo

**Fig. 7:**
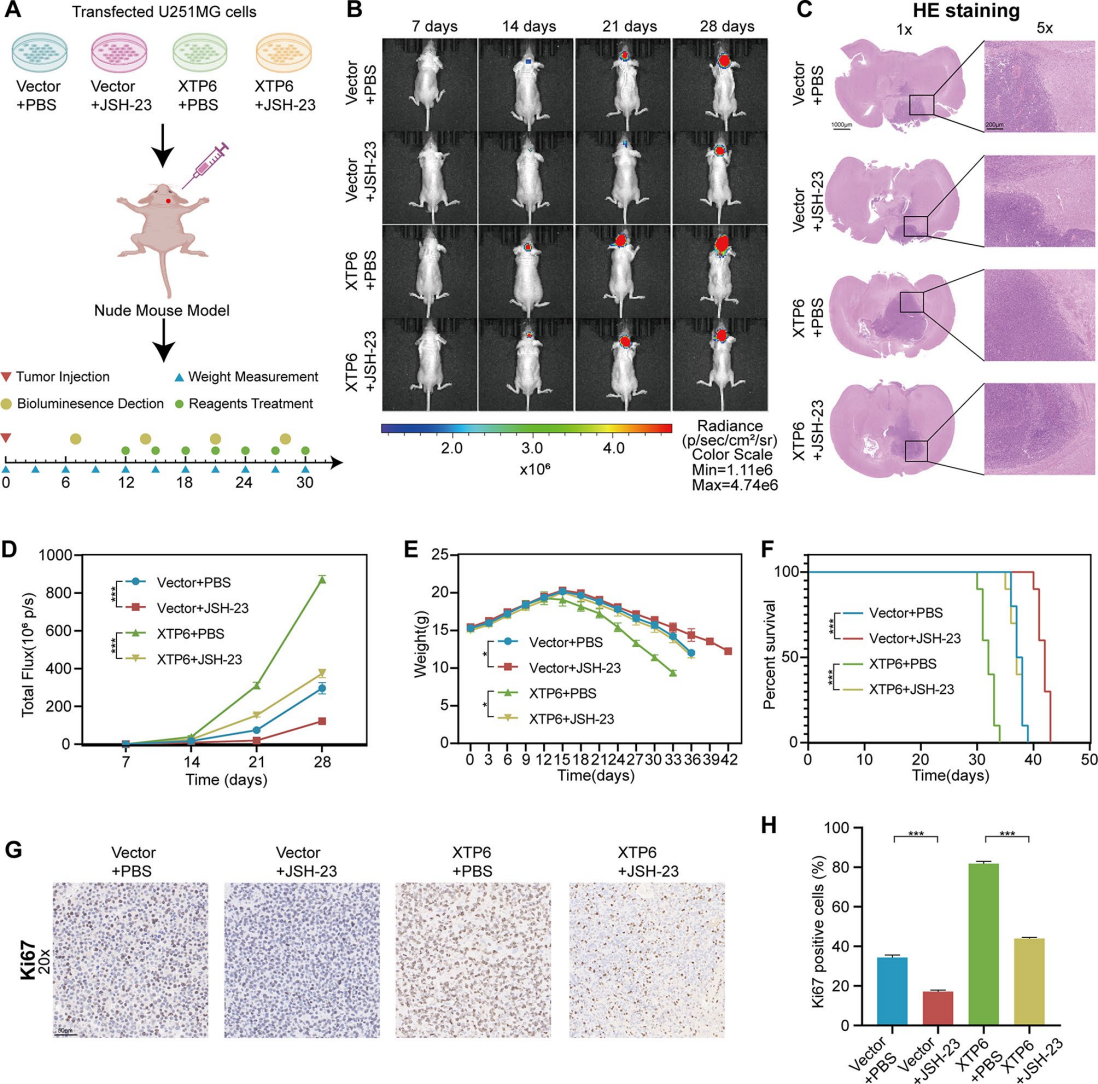
Inhibition of the NF-κB signaling pathway mitigates the progression of GBM induced by XTP6 in vivo. (**A**) Formation of intracranial xenograft mouse model. (**B**-**C**) In vitro imaging and HE staining were performed to evaluate the fluorescence intensity and size of intracranial tumors in nude mice in each experimental group. (**D**) The line chart was employed to evaluate the difference in changes in total fluorescence intensity of nude mice in each experimental group. (**E**) The line chart depicted the difference in body weight changes of nude mice within each experimental group across different time points. (**F**) Survival analysis was conducted for mice bearing tumors in each experimental group. (**G-H**) IHC analysis was employed to evaluate the differential expression of Ki67 among tumor tissues from nude mice in each experimental group. (*P < 0.05, **P < 0.01, ***P < 0.001)

Considering the critical role of XTP6 in maintaining the activation of the NF-κB signaling pathway for GBM progression, we proceeded to investigate whether inhibiting NF-κB signaling pathway could prevent the XTP6-driven GBM progression through constructing in vivo model (Fig. 7A). Overexpression of XTP6 facilitated tumor development in subcutaneous tumor models, while administration of JSH-23 markedly diminished the tumorigenic effects induced by XTP6 (Fig. 7B-D). Furthermore, it was observed that treatment with JSH-23 decelerated weight loss and extended the survival time of mice bearing tumors transduced with XTP6 (Fig. 7E, F). In addition, administering JSH-23 significantly diminished Ki-67 expression in GBM tissues that overexpressed XTP6, in comparison to those treated with PBS (Fig. 7G, H). These results verified that inhibiting the NF-κB signaling pathway could counteract the progression of GBM mediated by XTP6 in vivo.

## Discussion

GBM is recognized as the most common and aggressive primary brain tumor affecting adults, distinguished by its considerable heterogeneity and invasive growth [39, 40]. Despite progress in treatment strategies over recent decades, GBM patients still face a limited median survival time with current standard therapies [41, 42]. Consequently, it is imperative to unravel the molecular underpinnings that propel the advancement of GBM and to uncover substantiated targets for novel therapeutic interventions.

The identification of lncRNAs, defined as non-coding RNA molecules longer than 200 nucleotides, has opened novel avenues for comprehending the mechanisms underlying cancer initiation and progression [43–45]. LncRNAs exhibit diverse biological functions based on their distinct locations within the cell. Within the cell nucleus, lncRNAs typically participate in transcriptional regulation, influencing chromatin structure and gene expression control. For instance, they can modulate gene expression by promoting or inhibiting the transcriptional activity of specific genes. This regulation may involve gene silencing or activation, achieved through direct interactions with chromatin-associated proteins or by affecting the activity of RNA polymerase [12, 46, 47]. Within the cytoplasm, lncRNAs mainly exert their effects at the post-transcriptional stage, roles that encompass modulating mRNA stability, overseeing protein production, and facilitating the processing and transportation of RNA. These roles are facilitated through interactions with RNA-binding proteins or other molecules, thereby affecting protein production and intracellular signaling transmission [48–50]. Therefore, we deeply explored the biological roles of lncRNA in GBM in this research.

Firstly, the results of bioinformatics analysis revealed that XTP6 is overexpressed in GBM, and higher expression levels of XTP6 are connected with poorer prognosis in GBM patients. Additionally, we observed that XTP6 is elevated in both GBM tissues and cells, with presence in both the cytoplasm and nucleus, predominantly localized within the cytoplasm. Hence, XTP6, functioning as an oncogenic factor, could be pivotal in the aggressive advancement of GBM. Our findings indicated that silencing XTP6 markedly suppressed the malignant progression of GBM both in vitro and in vivo, whereas overexpression of XTP6 exerted converse impacts.

Subsequently, proteins pulled down by XTP6 in RNA pull-down assays were identified through mass spectrometry, which suggested that XTP6 most likely binds to NDH2. This interaction was further confirmed through RIP assays. NDH2 is an RNA helicase involved in genomic stability, transcription, and the regulation of DNA replication [51]. Numerous investigations have demonstrated that NDH2 is significantly involved in the onset, development, and cellular signaling pathways of cancer [52–55]. Moreover, through western blotting, ChIRP, ChIP, and dual-luciferase reporter assays, we confirmed that XTP6 recruits NDH2 to costruct a DNA-RNA triplex with the IκBα promoter. This interaction leads to the suppression of IκBα transcription by mediating H3K27me3 methylation, thereby activating the NF-κB signaling pathway. Therefore, XTP6 has been recognized as a functional binding partner of NDH2, regulating the NF-κB signaling pathway through an innovative mechanism that may be critical for the progression of GBM. Consequently, XTP6 has been recognized as an operative interacting partner of NDH2, orchestrating the NF-κB signaling pathway via an innovative mechanism potentially vital for the advancement of GBM.

Furthermore, our western blotting assays confirmed that c-myc is a crucial downstream regulatory factor of the NF-κB signaling pathway. Eventually, the results of ChIP assays and dual-luciferase reporter gene assays demonstrated that c-myc acts as an upstream transcription factor for XTP6, thereby influencing XTP6 expression. Hence, XTP6 sustains the activation of the NF-κB signaling pathway by establishing a positive feedback loop with c-myc.

**Fig. 8:**
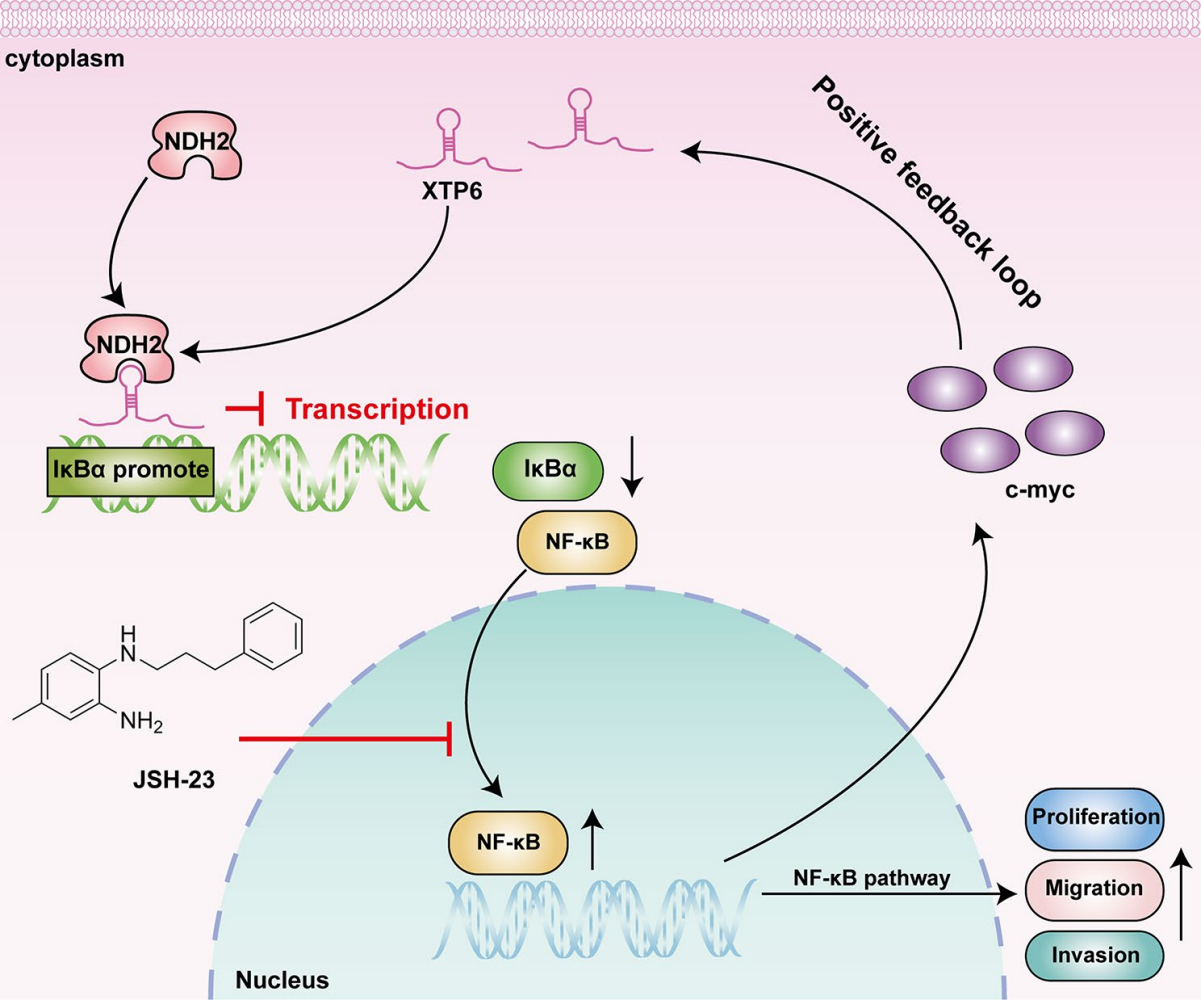
Schematic diagram of the molecular mechanism of this study

In summary, our findings suggest that the c-myc/XTP6/NDH2/NF-κB positive feedback loop can facilitate the malignant progression of GBM (Fig. 8), offering underlying new therapeutic targets for GBM patients.

## Conclusion

In conclusion, the results suggest that the c-myc/XTP6/NDH2/NF-κB positive feedback loop is crucial in facilitating the malignant progression of GBM. Comprehending the pivotal function of XTP6 in GBM and its involvement in activating the NF-κB signaling pathway enhances our understanding of the molecular mechanisms driving GBM progression. This insight could pave the way for creating innovative therapeutic agents for GBM patients.

### Electronic supplementary material

Below is the link to the electronic supplementary material.

Supplementary Material 1

Supplementary Material 2

Supplementary Material 3

Supplementary Material 4

Supplementary Material 5

Supplementary Material 6

Supplementary Material 7

Supplementary Material 8

Supplementary Material 9

Supplementary Material 10

Supplementary Material 11

## Data Availability

The data analyzed in this research can be found in the CGGA (http://www.cgga.org.cn/) and GEO (https://www.ncbi.nlm.nih.gov/gds) websites.

## Abbreviations

lncRNAs: Long Noncoding RNAs
RIP: RNA Immunoprecipitation
ChIRP: Chromatin Isolation by RNA Purification
ChIP: Chromatin Immunoprecipitation
GBM: Glioblastoma Multiforme
IκB: The Inhibitory κB
IKK: IκB Kinase
IκBα: Inhibitory κBα
PCTs: Paracancerous Tissues
IHC: Immunohistochemistry
RNA-ISH: RNA In Situ Hybridization
H-score: Histochemical Score
NHA: Normal Human Astrocyte
FISH: Fluorescence In Situ Hybridization
PBS: Phosphate-Buffered Saline
MS: Mass Spectrometry
RIPA: Radioimmunoprecipitation Assay Buffer
PVDF: Polyvinylidene Fluoride
HE: Hematoxylin-Eosin
OS: Overall Survival
NBT: Normal Brain Tissues
