# Supplementary material for: Positive feedback loop of c-myc/XTP6/NDH2/NF-κB to promote malignant progression in glioblastoma
Source: J Exp Clin Cancer Res. 2024 Jul 5;43:187. doi: 10.1186/s13046-024-03109-5 (PMC11225266; doi:10.1186/s13046-024-03109-5)
Supplement: Supplementary file 1 — Supplementary Material 1 [file 13046_2024_3109_MOESM1_ESM.docx]

**Table S1. Primer of qRT-PCR.**

| **Gene (human)** | **Forward（5′-3′）** | **Reverse（5′-3′）** |
| --- | --- | --- |
| XTP6 | GCGGAGGTGAAGTGAACTTAGA | CTCCTAAGCAGGACCCGTATT |
| NDH2 | CGAACCATCTCAGCGACAAAA | TGAGGTCCATGCTTATTTGCTC |
| KRT1 | AGAGTGGACCAACTGAAGAGT | ATTCTCTGCATTTGTCCGCTT |
| KRT10 | ATGTCTGTTCGATACAGCTCAAG | CTCCACCAAGGGAGCCTTTG |
| ADAR | CTGAGACCAAAAGAAACGCAGA | GCCATTGTAATGAACAGGTGGTT |
| KRT9 | GGGGCCGATTCAGCTCTTC | CTACTGGCACTAAAACCACCC |
| KRT2 | GCTGGCCCTAGATGTGGAGA | CGGAACTGGACCCTCTACC |
| IL-8 | GGGTGGAAAGGTTTGGAGTAT | TAGGACAAGAGCCAGGAAGAA |
| IκBα | TGGTCAGTGCCTTTTCTTCAT | GGAGTACGAGCAGATGGTCAA |
| c-myc | GCGACTCTGAGGAGGAA | TGCGTAGTTGTGCTGATG |
| TGF-β | TGCCTTCAGCAGAGTGAAGA | GTCTTGGTTCTCAGCTTGGG |
| STAT3 | CAGCAGCTTGACACACGGTA | AAACACCAAAGTGGCATGTGA |
| IGFBP3 | AGAGCACAGATACCCAGAACT | GGTGATTCAGTGTGTCTTCCATT |
| PAI-1 | ACCGCAACGTGGTTTTCTCA | TTGAATCCCATAGCTGCTTGAAT |
| P50 | AACAGAGAGGATTTCGTTTCCG | TTTGACCTGAGGGTAAGACTTCT |
| P65 | GAAGAGCAGCGTGGGGACTAC | CAAAGATGGGATGAGAAAGGACAGG |
| c-Rel | GCAGAGGGGAATGCGTTTTAG | AGAAGGGTATGTTCGGTTGTTG |
| RELA | ATGTGGAGATCATTGAGCAGC | CCTGGTCCTGTGTAGCCATT |
| GAPDH | AACGGATTTGGTCGTATTGGG | GGCAACAATATCCACTTTACCAGA |
