# Supplementary material for: Positive feedback loop of c-myc/XTP6/NDH2/NF-κB to promote malignant progression in glioblastoma
Source: J Exp Clin Cancer Res. 2024 Jul 5;43:187. doi: 10.1186/s13046-024-03109-5 (PMC11225266; doi:10.1186/s13046-024-03109-5)
Supplement: Supplementary file 2 — Supplementary Material 2 [file 13046_2024_3109_MOESM2_ESM.docx]

**Table S2. Gene knockdown target sequence information.**

| **Gene (human)** | **Target sequence information** |
| --- | --- |
| si-XTP6#1 | GCGGAGGTGAAGTGAACTTAG |
| si-XTP6#2 | GCCAGTGTCTAAACTCCAAAC |
| si-NDH2#1 | AGCUCGUCUAAACCAAUAU |
| si-NDH2#2 | GCCACAUAAACCUGAAUAA |
| si-c-myc#1 | GCTTCACCAACAGGAACTATG |
| si-c-myc#2 | GGAAACGACGAGAACAGTTGA |
