# Supplementary material for: Positive feedback loop of c-myc/XTP6/NDH2/NF-κB to promote malignant progression in glioblastoma
Source: J Exp Clin Cancer Res. 2024 Jul 5;43:187. doi: 10.1186/s13046-024-03109-5 (PMC11225266; doi:10.1186/s13046-024-03109-5)
Supplement: Supplementary file 3 — Supplementary Material 3 [file 13046_2024_3109_MOESM3_ESM.docx]

**Table S4. Probes of ChIRP assays.**

| **Probes** | **Sequence information (5′ to 3′)** |
| --- | --- |
| XTP6#1_odd | CCATATACAAGGTCTCATTGATACATTCCGT |
| XTP6#2_odd | GTTATAGAACAGGATAAGGCAGGTCATCTC |
| XTP6#3_odd | TTGATATTGGGTAGAAGTCTGCTGGAATAG |
| XTP6#4_odd | AATTAATATTTCCAATATAACACCATTCCACAGAAA |
| XTP6#5_odd | AATTCACAACTCAGACTTATAGCTCCTTACTT |
| XTP6#1_even | TGTGACTCAATTCCTGAAGGTTCTGTTCTT |
| XTP6#2_even | TTTAAGGAGTGGTGAATTTGGATTGCCAGA |
| XTP6#3_even | GTTTCTCTTTAGTTTGTACTTAAATTGAACACTGAA |
| XTP6#4_even | CATGGAGGAAACCTGTGAAATAACCTAATAT |
| XTP6#5_even | TTTATTTAAGAATTAACCTCTATCGTTTACCTCCTTT |
