# Supplementary material for: Positive feedback loop of c-myc/XTP6/NDH2/NF-κB to promote malignant progression in glioblastoma
Source: J Exp Clin Cancer Res. 2024 Jul 5;43:187. doi: 10.1186/s13046-024-03109-5 (PMC11225266; doi:10.1186/s13046-024-03109-5)
Supplement: Supplementary file 4 — Supplementary Material 4 [file 13046_2024_3109_MOESM4_ESM.docx]

**Table S5. Primer of ChIP-qPCR.**

| **Gene (human)** | **Forward（5′-3′）** | **Reverse（5′-3′）** |
| --- | --- | --- |
| IκBα-ChIP | GGGCTCAAGAGATCCAC | GACATCGTTTGTGGATCAGGA |
|  | AGCGATCCTCCCAACTC | TTTTGGCTGGGTGTGG |
| XTP6-ChIP | AACATCACATTGCACTCTTC | TCTGCAGCAGCTATTCTG |
|  | CATTCTAAGCGAGGGC | GGCTCTGGGTTGCAAATATT |
|  | CCTAAAGTTAATGCCGTCAC | TCCCCCAAAAGTCTTTACCC |
|  | TCACATCACTCAAGAGCTGTGGA | TTGAAACATGTCGCAAGGA |
