# Supplementary material for: Positive feedback loop of c-myc/XTP6/NDH2/NF-κB to promote malignant progression in glioblastoma
Source: J Exp Clin Cancer Res. 2024 Jul 5;43:187. doi: 10.1186/s13046-024-03109-5 (PMC11225266; doi:10.1186/s13046-024-03109-5)
Supplement: Supplementary file 5 — Supplementary Material 5 [file 13046_2024_3109_MOESM5_ESM.docx]

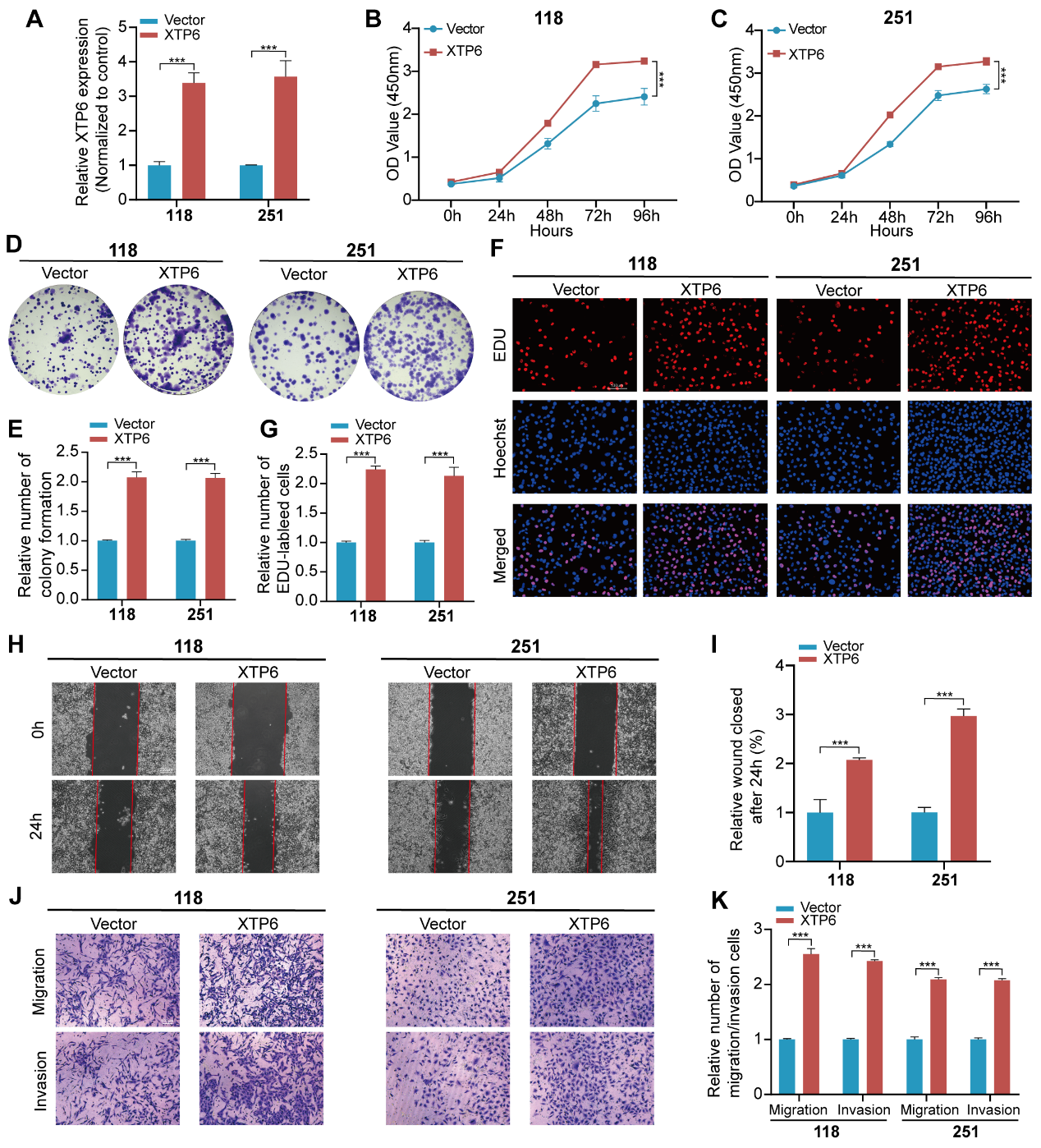


**Fig. S1** Overexpression of XTP6 promotes proliferation, migration, and invasion of GBM cells. (**A**) qRT-PCR assays verified the efficiencies of XTP6 overexpression in U118MG and U251MG cells. (**B**-**C**) The cell viability of XTP6-cDNA-transfected U118MG (**B**) and U251MG (**C**) cells by CCK-8 assays. (**D**-**E**) Effect of XTP6 overexpression on colony formation was counted in U118MG and U251MG cells (**D**). Histogram analysis revealed the mean ± standard deviation (SD) of colony counts across three independent experiments (**E**). (**F**-**G**) Representative images (**F**) and histogram analysis (**G**) showed the outcomes of EdU assays following the overexpression of XTP6 in U118MG and U251MG cells. (**H**-**I**) Representative images (**H**) and histogram analysis (**I**) illustrated the results of wound healing assays after overexpression of XTP6 in U118MG and U251MG cells. (**J**-**K**) Representative images (**J**) and histogram analysis (**K**) indicated the effects of XTP6 overexpression on Transwell assays in U118MG and U251MG cells. (**P* < 0.05, ***P* < 0.01, ****P* < 0.001)
