# Supplementary material for: Positive feedback loop of c-myc/XTP6/NDH2/NF-κB to promote malignant progression in glioblastoma
Source: J Exp Clin Cancer Res. 2024 Jul 5;43:187. doi: 10.1186/s13046-024-03109-5 (PMC11225266; doi:10.1186/s13046-024-03109-5)
Supplement: Supplementary file 6 — Supplementary Material 6 [file 13046_2024_3109_MOESM6_ESM.docx]

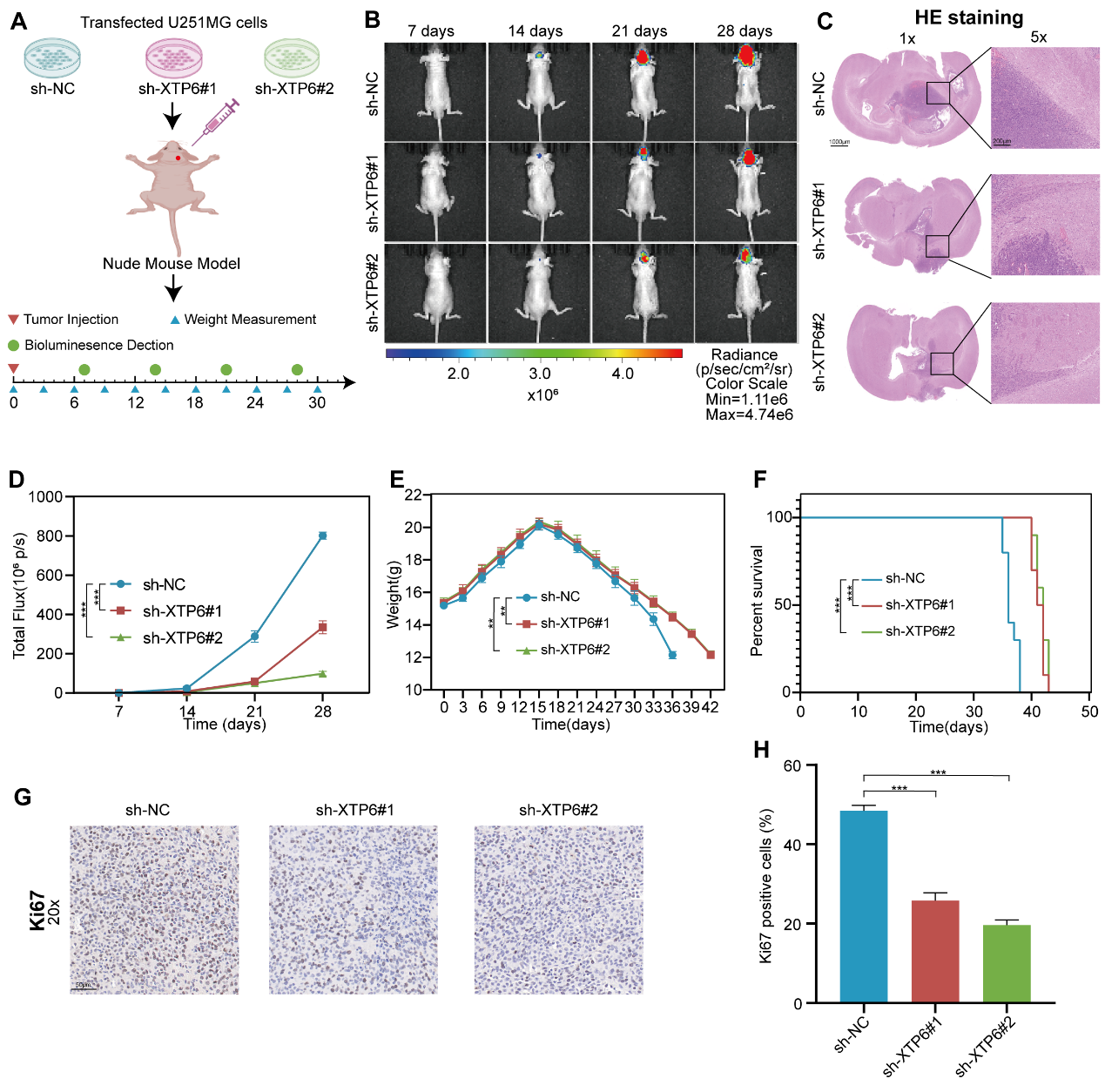


**Fig. S2** Knockdown of XTP6 inhibits the formation of GBM *in vivo*. (**A**) Construction of intracranial xenograft mouse model. (**B**-**C**) *In vitro* imaging and HE staining were employed to assess the fluorescence intensity and size of intracranial tumors in nude mice. (**D**) The line chart was utilized to evaluate the difference in changes in total fluorescence intensity of nude mice between control and sh-XTP6 groups. (**E**) The line chart displayed the difference in body weight changes of nude mice within control and sh-XTP6 groups across different time points. (**F**) Survival analysis was performed for mice bearing tumors in control and sh-XTP6 groups. (**G-H**) IHC analysis was conducted to detect the differential expression of Ki67 among tumor tissues from nude mice in control and sh-XTP6 groups group. (**P* < 0.05, ***P* < 0.01, ****P* < 0.001)
