# Supplementary material for: Positive feedback loop of c-myc/XTP6/NDH2/NF-κB to promote malignant progression in glioblastoma
Source: J Exp Clin Cancer Res. 2024 Jul 5;43:187. doi: 10.1186/s13046-024-03109-5 (PMC11225266; doi:10.1186/s13046-024-03109-5)
Supplement: Supplementary file 7 — Supplementary Material 7 [file 13046_2024_3109_MOESM7_ESM.docx]

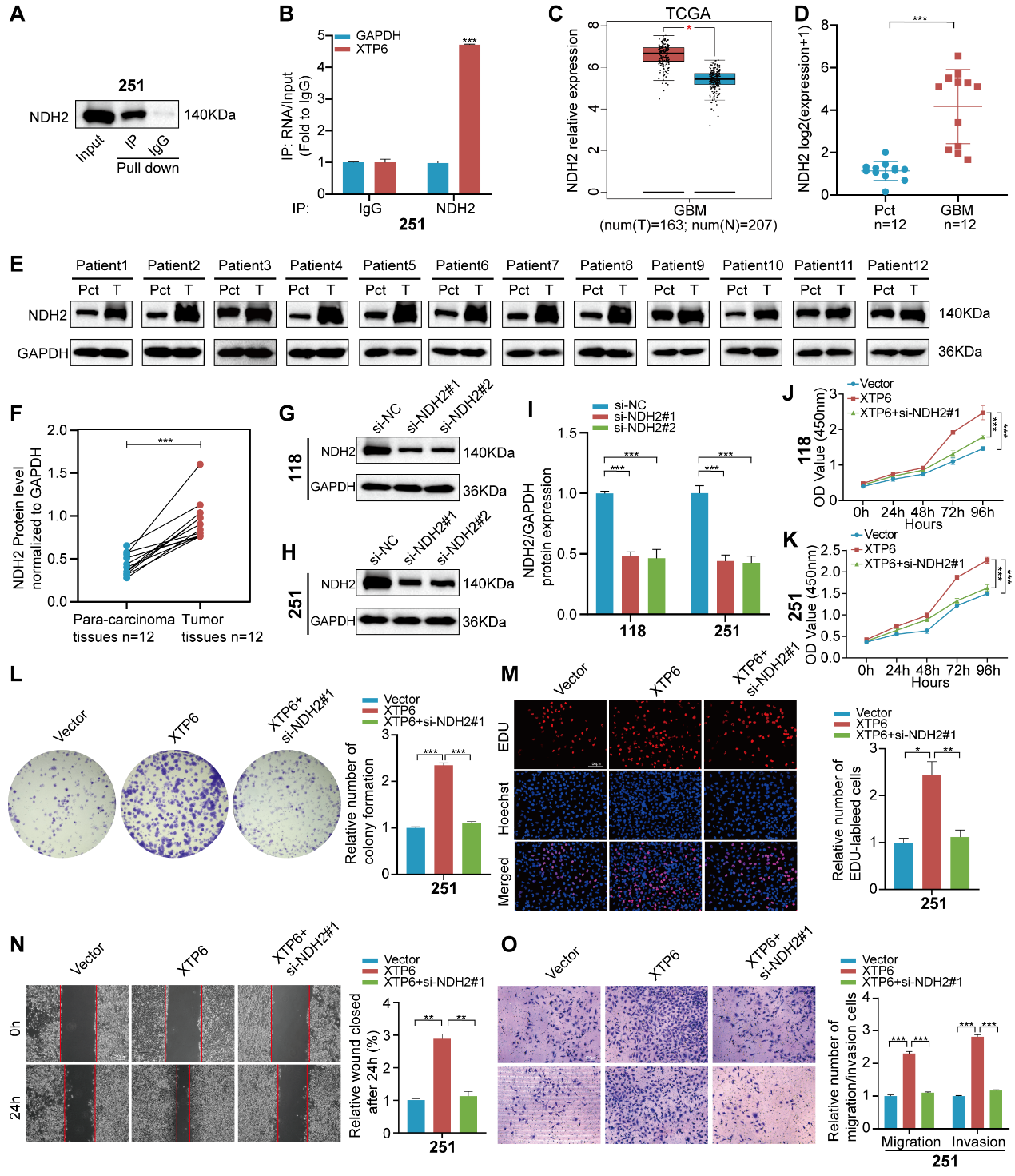


**Fig. S3** NDH2 is required for XTP6-induced GBM progression. (**A**) Western blotting analysis of proteins obtained by XTP6 probes, suggesting that XTP6 interacts with NDH2 in U251MG cells. (**B**) RIP assays indicated that XTP6 bound to NDH2 in U251MG cells. (**C**) The prediction results of the GEPIA website showed that NDH2 was highly expressed in GBM. (**D**) qRT-PCR analysis indicated that the expression of NDH2 in GBM tissue was significantly higher than that in the corresponding PCTs. (**E-F**) The results of western blotting analysis suggested that the expression of NDH2 protein in GBM tissue was significantly higher than that in the corresponding PCTs. (**G-I**) Western blotting analyses were employed to check the efficiencies of NDH2 knockdown in U118MG (**G**) and U251MG (**H**) cells. Histogram analysis displayed the results of western blotting assays (**I**). (**J**-**K**) CCK-8 assays indicated that knockdown of NDH2 partly reversed the impact of XTP6 overexpression in U118MG (**J**) and U251MG (**K**) cells. (**L-O**) Colony formation (**L**), EdU (**M**), Wound healing (**N**), and Transwell (**O**) assays demonstrated that knockdown of NDH2 partly reversed the impact of XTP6 overexpression in U251MG cells. (**P* < 0.05, ***P* < 0.01, ****P* < 0.001)
