# Supplementary material for: Positive feedback loop of c-myc/XTP6/NDH2/NF-κB to promote malignant progression in glioblastoma
Source: J Exp Clin Cancer Res. 2024 Jul 5;43:187. doi: 10.1186/s13046-024-03109-5 (PMC11225266; doi:10.1186/s13046-024-03109-5)
Supplement: Supplementary file 8 — Supplementary Material 8 [file 13046_2024_3109_MOESM8_ESM.docx]

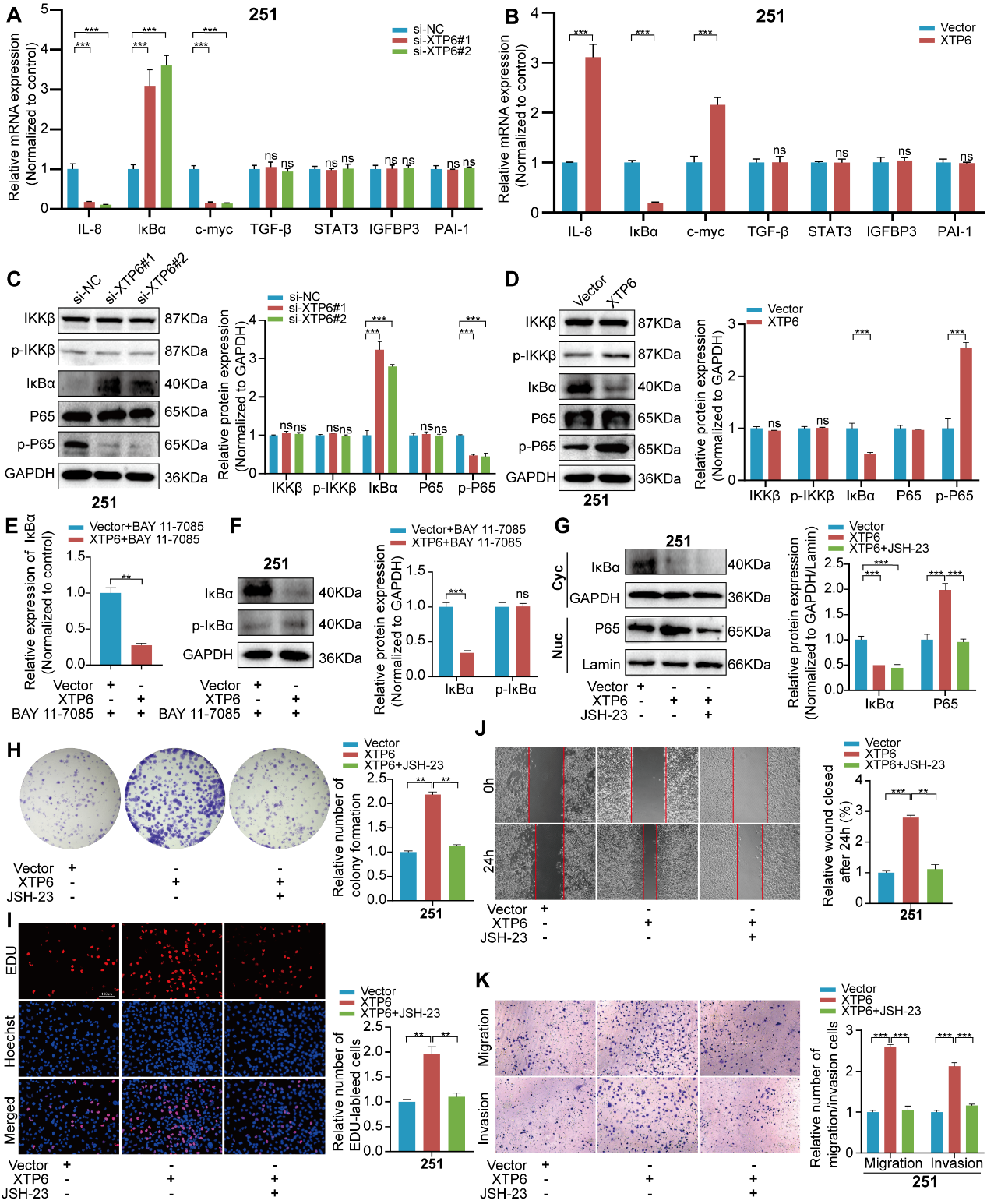


**Fig. S4** XTP6 activates the NF-κB signaling pathway through downregulating the IκBα expression in U251MG cells. (**A**-**B**) The expression of genes within the NF-κB signaling pathway was assessed via qRT-PCR assay in U251MG cells subjected to knockdown (**A**) or overexpression (**B**) of XTP6. (**C**-**D**) Western blotting analysis revealed alterations in protein levels associated with the NF-κB signaling pathway following the knockdown (**C**) or overexpression (**D**) of XTP6 in U251MG cells. (**E**-**F**) qRT-PCR and Western blotting analyses demonstrated that treatment with BAY 11–7085 led to a downregulation of IκBα at both mRNA (**E**) and protein (**F**) levels in U251MG cells mediated by XTP6. (**G**) Western blotting assays suggested that JSH-23 can reverse the translocation of P65 mediated by XTP6 in U251MG cells. (**H-K**) Colony formation (**H**), EdU (**I**), Wound healing (**J**), and Transwell (**K**) assays indicated that JSH-23 can reverse the effects of XTP6-overexpressing U251MG cells. (**P* < 0.05, ***P* < 0.01, ****P* < 0.001)
