# Supplementary material for: Positive feedback loop of c-myc/XTP6/NDH2/NF-κB to promote malignant progression in glioblastoma
Source: J Exp Clin Cancer Res. 2024 Jul 5;43:187. doi: 10.1186/s13046-024-03109-5 (PMC11225266; doi:10.1186/s13046-024-03109-5)
Supplement: Supplementary file 10 — Supplementary Material 10 [file 13046_2024_3109_MOESM10_ESM.docx]

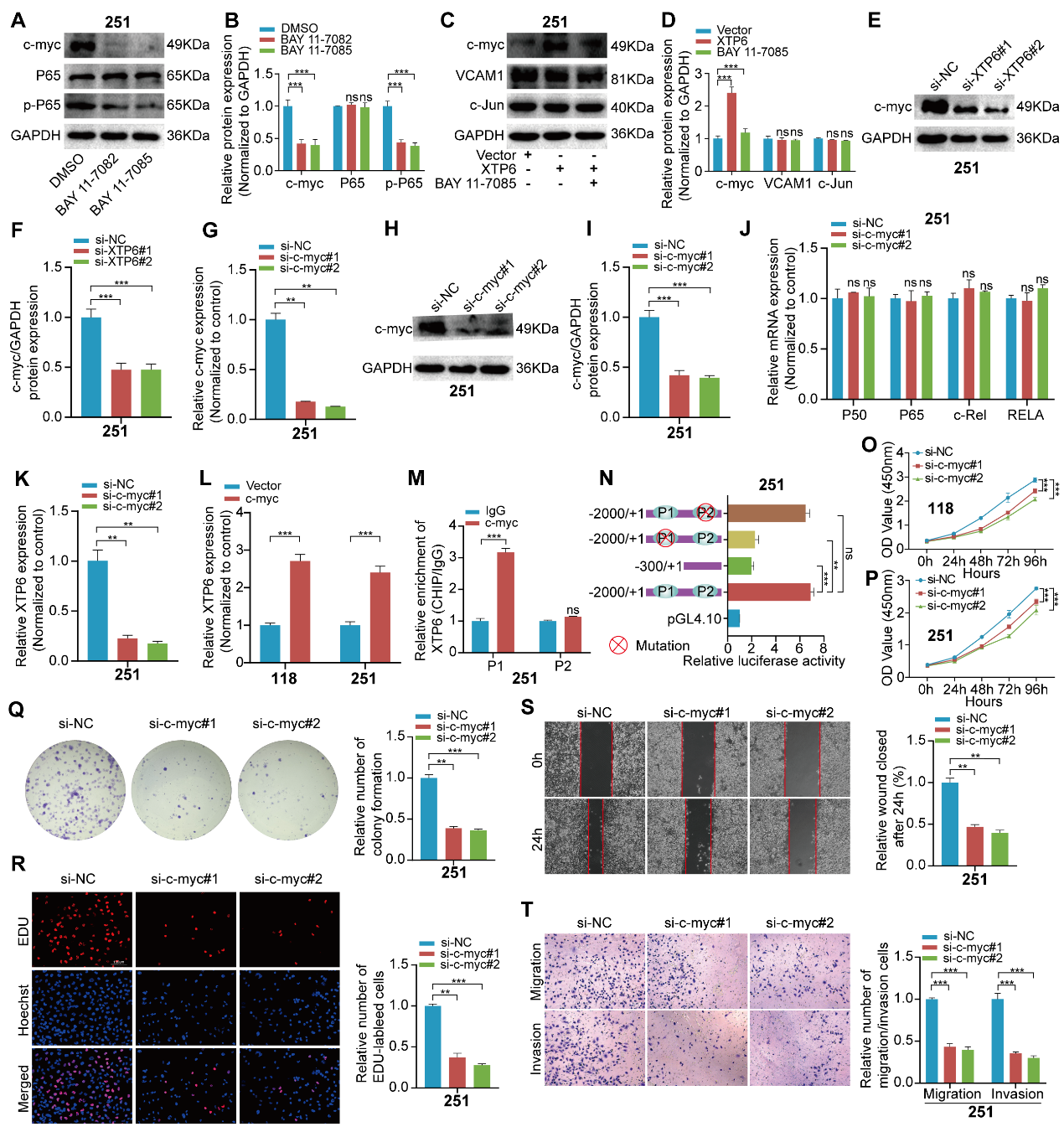


**Fig. S6** XTP6 promotes sustained activation of the NF-κB signaling pathway by establishing a positive feedback loop with c-myc in U251MG cells. (**A**-**B**) Western blotting analysis showed that administering NF-κB inhibitors, specifically BAY 11-7082 and BAY 11-7085, led to a reduction in the expression levels of c-myc and p-p65 in U251MG cells. (**C**-**D**) Western blotting analysis indicated that after treating XTP6-overexpressing U251MG cells with BAY 11-7085, the expression levels of c-myc, VCAM1, and C-Jun were assessed 72 hours post-treatment. (**E**-**F**) Western blotting analysis was employed to assess the expression of c-myc after XTP6 knockdown in U251MG cells. (**G-I**) qRT-PCR (**G**) and Western blotting (**H, I**) analyses were utilized to inspect the efficiencies of c-myc knockdown in U251MG cells. (**J**) qRT-PCR assays revealed that the depletion of c-myc did not affect the expression levels of P50, P65, c-Rel, and RELA in U251MG cells. (**K**) qRT-PCR analysis demonstrated that c-myc depletion led to a decrease in XTP6 expression in U251MG cells. (**L**) qRT-PCR analysis revealed that overexpression of c-myc resulted in an increase in XTP6 expression in U118MG and U251MG cells. (**M**) ChIP-qPCR analysis was performed in U251MG cells. (**N**) Luciferase reporter assays demonstrated that the depletion of P1 can lead to a reduction in the transcriptional activity of the XTP6 promoter in U251MG cells. (**O**-**P**) The cell viability of si-c-myc-transfected U118MG (**O**) and U251MG (**P**) cells by CCK-8 assays. (**Q-T**) Colony formation (**Q**), EdU (**R**), Wound healing (**S**), and Transwell (**T**) assays showed that knockdown of c-myc can inhibit the proliferation, migration and invasion of U251MG cells. (**P* < 0.05, ***P* < 0.01, ****P* < 0.001)
