# Supplementary material for: Positive feedback loop of c-myc/XTP6/NDH2/NF-κB to promote malignant progression in glioblastoma
Source: J Exp Clin Cancer Res. 2024 Jul 5;43:187. doi: 10.1186/s13046-024-03109-5 (PMC11225266; doi:10.1186/s13046-024-03109-5)
Supplement: Supplementary file 11 — Supplementary Material 11 [file 13046_2024_3109_MOESM11_ESM.docx]

**Table S3. Antibodies and agents of experiments.**

| **Product** | **No. of Catalogue** | **Supplier** |
| --- | --- | --- |
| NDH2 antibody | 17721-1-AP | Proteintech |
| P65 antibody | 8242 | Cell Signaling Technology |
| p-P65 antibody | 3033 | Cell Signaling Technology |
| c-myc antibody | 13987 | Cell Signaling Technology |
| IKKβ antibody | 8943 | Cell Signaling Technology |
| p-IKKβ antibody | 2078 | Cell Signaling Technology |
| IκBα antibody | 4812 | Cell Signaling Technology |
| p-IκBα antibody | 2859 | Cell Signaling Technology |
| Lamin antibody | 12987-1-AP | Proteintech |
| H3K27me3 | 9733 | Cell Signaling Technology |
| EZH2 | 5246 | Cell Signaling Technology |
| H3K9me3 | 13969 | Cell Signaling Technology |
| H3K4me3 | 9751 | Cell Signaling Technology |
| VCAM1 antibody | ab134047 | Abcam |
| c-Jun antibody | 9165 | Cell Signaling Technology |
| Secondary antibody (goat anti-mouse) | 31430 | Thermo Fisher |
| Secondary antibody (goat anti-rabbit) | 31460 | Thermo Fisher |
| BAY 11–7082 | HY-13453 | MedChemExpress |
| BAY 11–7085 | HY-10257 | MedChemExpress |
| JSH-23 | HY-13982 | MedChemExpress |
